# Luminescence and Dielectric Switchable Properties of a 1D (1,1,1-Trimethylhydrazinium)PbI_3_ Hybrid Perovskitoid

**DOI:** 10.1021/acs.inorgchem.2c03287

**Published:** 2022-12-15

**Authors:** Jan. A. Zienkiewicz, Karolina Kałduńska, Katarzyna Fedoruk, Antonio J. Barros dos Santos, Mariusz Stefanski, Waldeci Paraguassu, Tadeusz M. Muzioł, Maciej Ptak

**Affiliations:** † Institute of Low Temperature and Structure Research, Polish Academy of Sciences, Okólna 2, 50-422Wrocław, Poland; ‡ Institute of Physics, Wrocław University of Science and Technology, Wybrzeże Wyspiańskiego 27, 50-370Wrocław, Poland; § Department of Biomedical and Polymer Chemistry, Faculty of Chemistry, Nicolaus Copernicus University in Toruń, Gagarina 7, 87-100Toruń, Poland; ∥ Department of Physics, Federal University of Pará, Campus do Guamá, Rua Augusto Corrêa 01, Belém, 66075110Pará, Brazil; ⊥ Department of Inorganic and Coordination Chemistry, Faculty of Chemistry, Nicolaus Copernicus University in Toruń, Gagarina 7, 87-100Toruń, Poland

## Abstract

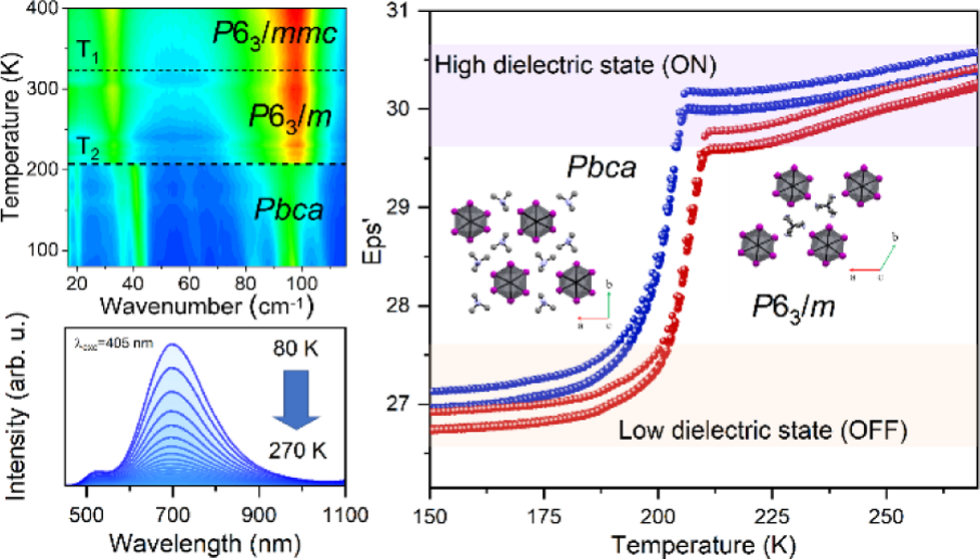

The synthesis and investigation of the physicochemical properties of a novel one-dimensional (1D) hybrid organic–inorganic perovskitoid templated by the 1,1,1-trimethylhydrazinium (Me_3_Hy^+^) cation are reported. (Me_3_Hy)[PbI_3_] crystallizes in the hexagonal *P*6_3_/*m* symmetry and undergoes two phase transitions (PTs) during heating (cooling) at 322 (320) and 207 (202) K. X-ray diffraction data and temperature-dependent vibrational studies show that the second-order PT to the high-temperature hexagonal *P*6_3_/*mmc* phase is associated with a weak change in entropy and is related to weak structural changes and different confinement of cations in the available space. The second PT to the low-temperature orthorhombic *Pbca* phase that corresponds to the high change in entropy and dielectric switching is associated with an ordering of the trimethylhydrazinium cations, re-arrangement and strengthening of hydrogen bonds, and slightly shifted lead-iodide octahedral chains. The high-pressure Raman data revealed two additional PTs, one between 2.8 and 3.2 GPa, related to the symmetry decrease, ordering of the cations, and inorganic chain distortion, and the other in the 6.4–6.8 GPa range related to the partial and reversible amorphization. Optical studies revealed that (Me_3_Hy)[PbI_3_] has a wide band gap (3.20 eV) and emits reddish-orange excitonic emission at low temperatures with an activation energy of 65 meV.

## Abstract 2

We present the synthesis and detailed physicochemical evaluation of a novel perovskitoid, 1,1,1-trimethylhydrazinium lead iodide. The compound undergoes two phase transformations, one of which is an order−disorder transformation associated with dielectric switching. At low temperatures, the material emits a reddish-orange luminescence, with temperature having a significant impact on intensity.

## Introduction

Lead halides are one of the most well-known and researched groups of hybrid organic–inorganic perovskites, particularly for their photovoltaic applications.^1,2^ They may exhibit interesting ferroelectric,^3−5^ ferroelastic,^6,7^ magnetic,^8^ second harmonic generation,^9^ switchable dielectric,^10^ or luminescence^8,10,11^ properties. While typical hybrid perovskites with a tolerance factor (TF) of 0.8 to 1 adopt a three-dimensional (3D) corner-sharing structure, compounds with higher TF are more likely to form one- or two-dimensional structures. Some of them have recently been called “perovskitoids.”

Stoumpos et al. introduced this new term in materials science to describe compounds having ABX_3_ stoichiometry and exclusively face-sharing connectivity between BX_6_ octahedra.^12^ The physicochemical properties of those hybrids (in oxide nomenclature termed 2H polytype) significantly differ from corner-sharing archetypical perovskites (3C polytype).^12^ Later, the term “perovskitoid” was extended to include structures similar to perovskite but with connectivity other than corner-sharing, such as face- or edge-sharing.^13^

Similar to perovskites, perovskitoids also have interesting physicochemical properties that are important for optoelectronics.^12−14^ One of them is the improvement of the application properties of perovskite solar cells (PSCs).^15−17^ Despite many advantages of the commonly used MAPbI_3_ and FAPbI_3_ (MA^+^ = methylammonium cation; FA^+^ = formamidinium cation), such as low-production costs and high-power conversion efficiency, PSCs have relatively low intrinsic stability. One of the suggested ways to boost photovoltaic properties of PSCs is the passivation of the surface with other 1D perovskitoids templated by bulky ammonium cations, like diethyl-(2-chloroethyl)ammonium,^16^ cyclohexylmethylammonium,^18^ or methyl-1,3-propanediammonium.^15^

In recent years, hybrids templated by the hydrazinium cation^19^ and its derivatives, such as methylhydrazinium^8−11,20^ or 1,1-dimethylhydrazinium,^21,22^ have received attention. Both cations were successfully employed to form 3D or two-dimensional (2D) perovskites in halide,^9−11^ hypophosphite,^8^ or formate frameworks.^21−23^

Despite the suggestions that partially substituting the MA^+^ in MAPbI_3_ with the 1,1,1-trimethylhydrazinium cation (Me_3_Hy^+^) would improve the photovoltaic properties and increase the intrinsic stability,^17^ there is no published structure and detailed physicochemical characterization of (Me_3_Hy)[PbI_3_]. As a result, we have decided to undertake methodological research on this perovskitoid in order to define the observed phase transitions (PTs), clarify their mechanisms in detail, and to characterize the optical and phonon properties of each phase. We have also performed high-pressure Raman measurements of the low-wavenumber mode region, which is believed to be particularly sensitive even to very subtle structural changes, in order to assess the stability and flexibility of (Me_3_Hy)[PbI_3_].

## Experimental Section

### Materials and Synthesis

Commercially available lead(II) iodide (99%, Sigma-Aldrich, US), 1,1,1-trimethylhydrazinium iodide (97%, Sigma-Aldrich, US), *N*,*N*-dimethylformamide (DMF) (99.8%, Sigma-Aldrich, US), dimethylsulfoxide (DMSO) (99.9%, Merck), and methyl acetate (99.5%, Sigma-Aldrich, US) were employed without additional purification.

The antisolvent method was used to synthesize (Me_3_Hy)[PbI_3_]. Lead(II) iodide and 1,1,1-trimethylhydrazinium iodide were dissolved in a mixture of DMF and DMSO (5:1). This solution was placed in a glass vial with a loose top, which was then placed in a bigger vial containing methyl acetate (antisolvent). After 5 days, yellow crystals were extracted, filtered from the mother solution, and dried at room temperature in air. The phase purity of crystals was validated by the good agreement of the powder X-ray diffraction (PXRD) pattern with the simulated one based on the single-crystal data (Figure S1).

### X-ray Diffraction

All of the data for (Me_3_Hy)[PbI_3_] were collected using an XtaLAB Synergy-S (Rigaku—Oxford Diffraction) four circle diffractometer with monochromatic and microfocus MoKα (λ = 0.71073 Å) radiation. Experiments were carried out for a single yellow crystal with dimensions 0.139 × 0.112 × 0.083 mm, at 215, 230, 260, 290, 310, 330, 350, and 375 K temperatures. The complete data sets were collected at 230 and 375 K. For other temperatures, the strategy implemented in CrysAlis was applied. The data sets were obtained and processed with CrysAlisPro software (CrysAlisPRO (ver. 41_64.104a), Oxford Diffraction/Agilent Technologies UK Ltd, Yarnton, England). Absorption correction was also taken into account in CrysAlisPro software. The structures were solved using SHELX2014 program packages by direct methods and refined with the full-matrix least-squares method on *F*^2^.^24^ Nonhydrogen atoms in all phases were refined anisotropically, and the positions of hydrogen atoms were idealized with geometry and constrained during refinement with an implemented model in SHELX. The final refinement cycles were performed with the EXTI command due to the very high absorption coefficient reaching ca. 20 mm^–1^. In the final model of the structure at 375 K, two hydrogen atoms from theNH_2_ group are missing. The figures of (Me_3_Hy)[PbI_3_] were prepared using Mercury 4.0 software.^25^

The powder data for low-temperature (LT) phase were collected using a Rigaku XtaLAB Synergy-S system and CuKα radiation (λ = 1.54056 Å). The data were reduced first in CrysAlisPro and then in EXPO2014. The cell parameters were found using the McMaille routine, and then the space group was determined. The structure was solved by direct methods followed by a resolution bias modification procedure, and the chain was identified. Fourier recycling resulted in the location of the organic cation. Hydrogen atoms were assigned to calculated positions. To keep the reasonable geometry of the Me_3_Hy^+^ cation, several restraints for bond lengths and valence angles were applied. In the final model, two nitrogen atoms from the NH_2_ group were missing.

The structural data have been deposited at the Cambridge Crystallographic Data Centre [2159778 for the room-temperature (RT) phase and 2159781 for the high-temperature (HT) phase].

### Thermal Properties

Heat capacity was measured using a Mettler Toledo DSC-1 calorimeter (DSC, differential scanning calorimetry) with a high resolution of 0.4 μW. Nitrogen was used as a purging gas, and the heating and cooling rate was 5 K min^–1^. The mass of the measured sample was 16.46 mg. The excess heat capacity associated with the PT was calculated by subtracting from the data a baseline representing the system variation in the absence of PTs.

Thermogravimetric analysis (TGA) was performed in the temperature range of 303–973 K using a PerkinElmer TGA 4000. The sample weighed 28.80 mg, and a 5 K min^–1^ heating rate was used. The atmosphere was pure nitrogen gas.

### Dielectric Properties

Dielectric measurements of the examined samples were carried out using a broad-band impedance Novocontrol Alpha analyzer. Since the obtained single crystals were not large enough to perform single-crystal dielectric measurements, a pellet was placed between two copper, flat electrodes of the capacitor with a gap of 0.5 mm. A sinusoidal voltage with an amplitude of 1 V and a frequency in the range of 1 Hz to 1 MHz was applied across the sample. The measurements were taken every 1 K in the temperature range of 140–360 K. The temperature stability of the samples was better than 0.1 K. The temperature was stabilized by means of nitrogen gas using the Novocontrol Quattro system.

### Vibrational Properties

The Raman spectra (3500–75 cm^–1^ range) of a polycrystalline sample were recorded at RT using a Bruker FT 100/S spectrometer and YAG:Nd laser excitation at 1064 nm. Temperature-dependent (80–400 K) Raman spectra of randomly oriented single crystals in the 3500–50 cm^–1^ range were measured using a Renishaw inVia Raman spectrometer with a confocal DM2500 Leica optical microscope, a thermoelectrically cooled CCD detector, and an Ar^+^ ion laser operating at 488 nm. A Linkam THMS600 stage was used to regulate the temperature.

A Labram Evolution spectrometer from Horiba equipped with a microscope was used to record the high-pressure Raman spectra. A solid-state 514.5 nm laser line was used for excitation. The spectral resolution was set to 2 cm^–1^. A diamond anvil cell Diacell μScopeDAC-RT(G) from Almax easyLab with a diamond of 0.4 mm culets was used to achieve high pressures. The sample was put into a 100 μm hole drilled in a stainless-steel gasket with a thickness of 200 μm using an electric discharge machine from Almax easyLab. The pressure transmitting medium was Nujol (mineral oil). The values of pressure were calculated using the shifts of the ruby R_1_ and R_2_ fluorescence lines.

Mid-infrared (IR) (4000–400 cm^–1^) and far-IR (500–50 cm^–1^) transmittance spectra were measured in the KBr disc and Nujol mull in a polyethylene plate, respectively, using a Nicolet iS50 FT-IR spectrometer with a resolution of 2 cm^–1^. The temperature-dependent IR spectra in the 4000–600 cm^–1^ range were collected using a Nicolet iN10 FTIR microscope and a Linkam THMS600 cryostat cell equipped with ZnSe windows.

### Optical Properties

The absorption measurement was performed in the back-scattering mode using an Agilent Cary 5000 spectrophotometer. Temperature-dependent emission spectra were measured with the Hamamatsu photonic multichannel analyzer PMA-12 equipped with a BT-CCD linear image sensor. The laser diode operating at 405 nm was applied as an excitation source. The temperature of the samples during emission measurements was controlled by a Linkam THMS600 heating/freezing stage. The luminescence decay profiles were recorded using a femtosecond laser (Coherent Model Libra).

## Results and Discussion

### Thermal Properties

**Figure 1:**
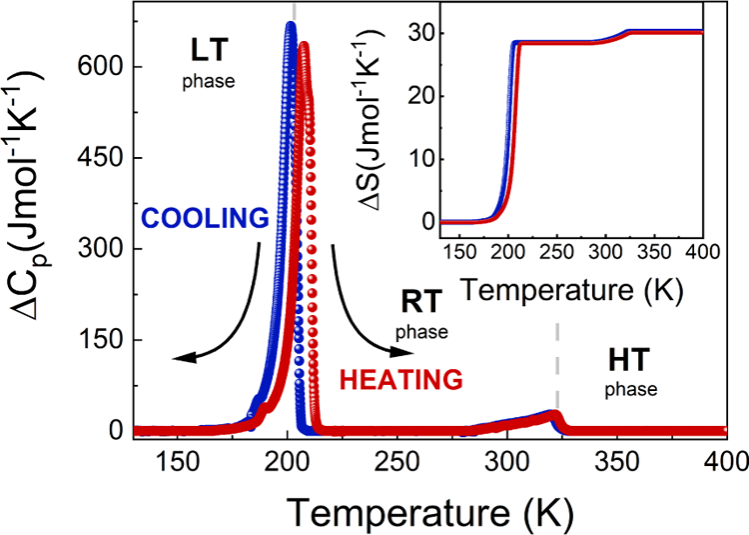
Temperature dependence of the excess heat capacity (Δ*C*_p_) obtained for heating (red) and cooling (blue); the inset shows the corresponding change of entropy (Δ*S*).

The PT behavior of (Me_3_Hy)[PbI_3_] was investigated before performing detailed crystal structure characterizations. The DSC curve demonstrates that (Me_3_Hy)[PbI_3_] undergoes two PTs during heating (cooling) at *T*_1_ = 322 (320) K and *T*_2_ = 207 (202) K (Figure S2). The Δ*C*_p_ peaks associated with the PT at *T*_1_ are asymmetric and do not show temperature hysteresis between heating and cooling cycles (Figure 1). The associated change in entropy (Δ*S*) varies continuously with temperature, indicating the second-order nature of PT at *T*_1_. The heat capacity peaks associated with PT at *T*_2_, on the other hand, are symmetrical, sharp, and with thermal hysteresis (5 K), indicating a first-order PT. This is further supported by the associated discontinuous change in Δ*S* (see Figure 1). The entropy changes were experimentally determined to be approximately 1.8 J mol^–1^ K^–1^ at *T*_1_ and 28.5 J mol^–1^ K^–1^ at *T*_2_.

According to X-ray diffraction (XRD) data (see further sections of this paper), the Me_3_Hy^+^ cations exhibit a sixfold disorder above *T*_2_. Assuming that the LT phase III is completely ordered, the PT at *T*_2_, separating the RT (II) and LT phases, according to the Boltzmann equation, would have a ratio of *N*_RT_/*N*_LT_ = 6, where *N*_*i*_ is the number of accessible microstates in the *i*th phase and *R* is the gas constant. As a result, the predicted entropy change at *T*_2_ would be around 15 J mol^–1^ K^–1^. Nearly twice as low as the experimental value may imply that this PT is more complicated than a simple order–disorder model would predict.

The estimate of the Δ*S* is higher than expected, suggesting that the disorder may be caused by both the movement of the cations and the disturbance of the entire skeleton. A further increase in entropy after going from RT to the HT phase is associated with subtle changes in unit cell parameters (see further sections). Furthermore, the total change of Δ*S* in (Me_3_Hy)[PbI_3_] is higher than recently reported for other known lead iodides, such as (methylhydrazinium)_2_[PbI_4_] (2D) (∼2.88 J mol^–1^ K^–1^), (*N*-methyldabconium)[PbI_3_] (1D) (∼4.3 J mol^–1^ K^–1^), and (*R*-2-methylpiperidinium)[PbI_3_] (1D) (∼29.5 J mol^–1^ K^–1^) analogues.^26−28^

Thermal stability has been used to evaluate the thermal stability of (Me_3_Hy)[PbI_3_]. The compound is stable up to roughly 573 K, according to the registered TGA curve shown in Figure S3. The (Me_3_Hy)[PbI_3_] decomposes in three stages, with weight losses of around 4.7, 19.2, and 44.8% at inflection points at 595, 650, and 830 K, respectively. According to the literature data, the first step of trimethylhydriazine decomposition leads to *N*-methylmetanimine and the release of methylamine,^29^ which is in good agreement with the registered weight loss. The residual *N*-methylmetaniminium lead iodide decomposes into *N*-methylmetanimine, PbI_2_, and HI in the subsequent stage at 650 K.^30^ The third stage involves the formation of HCN, CH_4_, and the I_2_ molecules, as well as residual of pure lead.^30^

### Dielectric Properties

**Figure 2:**
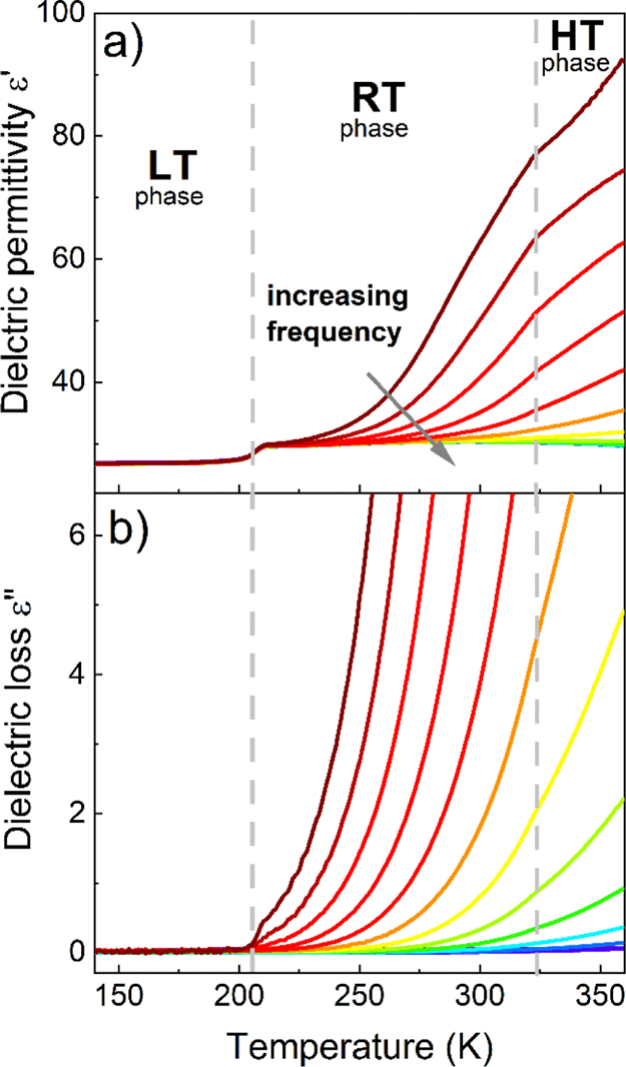
Temperature dependence of the dielectric permittivity (a) and dielectric loss (b) as a function of temperature measured for the pelletized (Me_3_Hy)[PbI_3_] during heating. The dashed lines represent the PT temperatures determined by DSC measurements.

In order to characterize the dielectric response of (Me_3_Hy)[PbI_3_], the complex dielectric permittivity as a function of frequency and temperature was investigated. Parts a and b of Figure 2 show the temperature dependences of the real ε′ and imaginary ε″ parts of the complex dielectric permittivity ε* = ε′ – iε″. During heating, after the PT at *T*_2_, ε′ first shows a step-like rise from 26.5 to 30.4. Such a sharp increase in the dielectric response related to a first-order PT is a common example of the phenomenon called dielectric switching.^21,28,31,32^ During continued heating, a high-frequency dispersion becomes dominant in the RT phase, which is most likely associated with ionic/electrical conductivity processes. Some hybrid perovskites, such as (FA)M^II^[H_2_PO_2_]_3_ (M^II^ = Cd, Mn), (MHy)_2_[PbI_4_], (MHy)[PbBr_3_] (MHy^+^ = methylhydrazinium cation), or [(CH_3_)_4_N]_4_Pb_3_Cl_10_, already demonstrated similar behaviors.^10,11,33,34^

**Figure 3:**
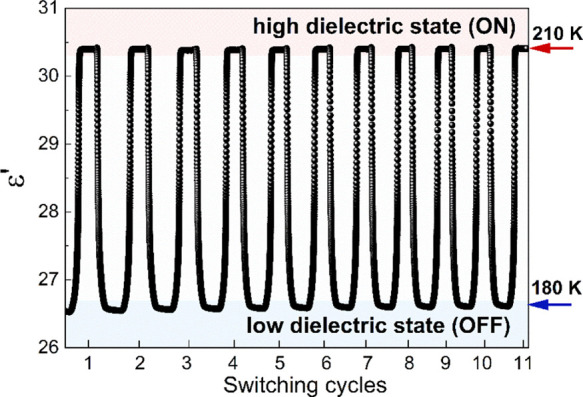
Several cycles of the temperature-induced dielectric switching of (Me_3_Hy)[PbI_3_] at a frequency of 0.05 MHz.

To better visualize the relaxation dynamics and exclude the contribution of electrode polarization, the frequency-dependent electric modulus (M* = 1/ε*) for several isotherms was analyzed for selected ones (Figure S4). The occurrence of the ionic/electrical conductivity process at HTs was revealed by isothermal complex electric modulus spectra with the characteristic bell (*M*″) and step (*M*′) shapes. Additionally, the dielectric permittivity was measured as a function of temperature and time to confirm the phase stability during many switches between on/off states. The material was left at the specified temperatures for 20 min following the switching cycles, which involved temperature changes between 180 and 210 K at a rate of 2 K min^–1^ (Figure 3). These results indicate that the examined material, when subjected to repeated temperature changes, is reversible and stable.

### Single-Crystal XRD

Depending on the temperature (Me_3_Hy)[PbI_3_] crystal exists in three phases, namely HT phase I, RT phase II, and LT phase III. The crystal data and refinement details for I and II phases are gathered in Table S1. Tables S2 and S3 list the geometric parameters for phases I and II, respectively. Crystals in the phase I adopt hexagonal *P*6_3_/*mmc* symmetry with unit cell volume *V*_HT_ = 658.44 Å^3^. The PT to the phase II is associated with a symmetry lowering to *P*6_3_/*m* and a reduction in the unit cell volume to *V*_RT_ = 635.57 Å^3^. The disappearance of *c* planes during PT at *T*_1_ (*P*6_3_/*mmc* → *P*6_3_/*m*) was confirmed by systematic absence analysis. Eventually, each structure-solving attempt with higher symmetry in phase II failed. In phase III, (Me_3_Hy)[PbI_3_] crystal alters symmetry to the orthorhombic system with the *Pbca* space group.

It should be highlighted that structural and symmetry modifications justify DSC data showing a strong peak at *T*_2_ and a weak one at *T*_1_. In the former case, this strong peak, with a corresponding high change in entropy, represents considerable symmetry and unit cell volume change. In the latter case, both parameters change slightly, and the crystal structure is very similar. Furthermore, repeated cycle diffraction experiments in the 215–375 K range showed that crystal-to-crystal PT at *T*_1_ is totally reversible and the crystal integrity is fully preserved. In contrast, the PT at *T*_2_ leads to permanent crystal disintegration and multidomain diffraction pattern (see powder experiments).

**Figure 4:**
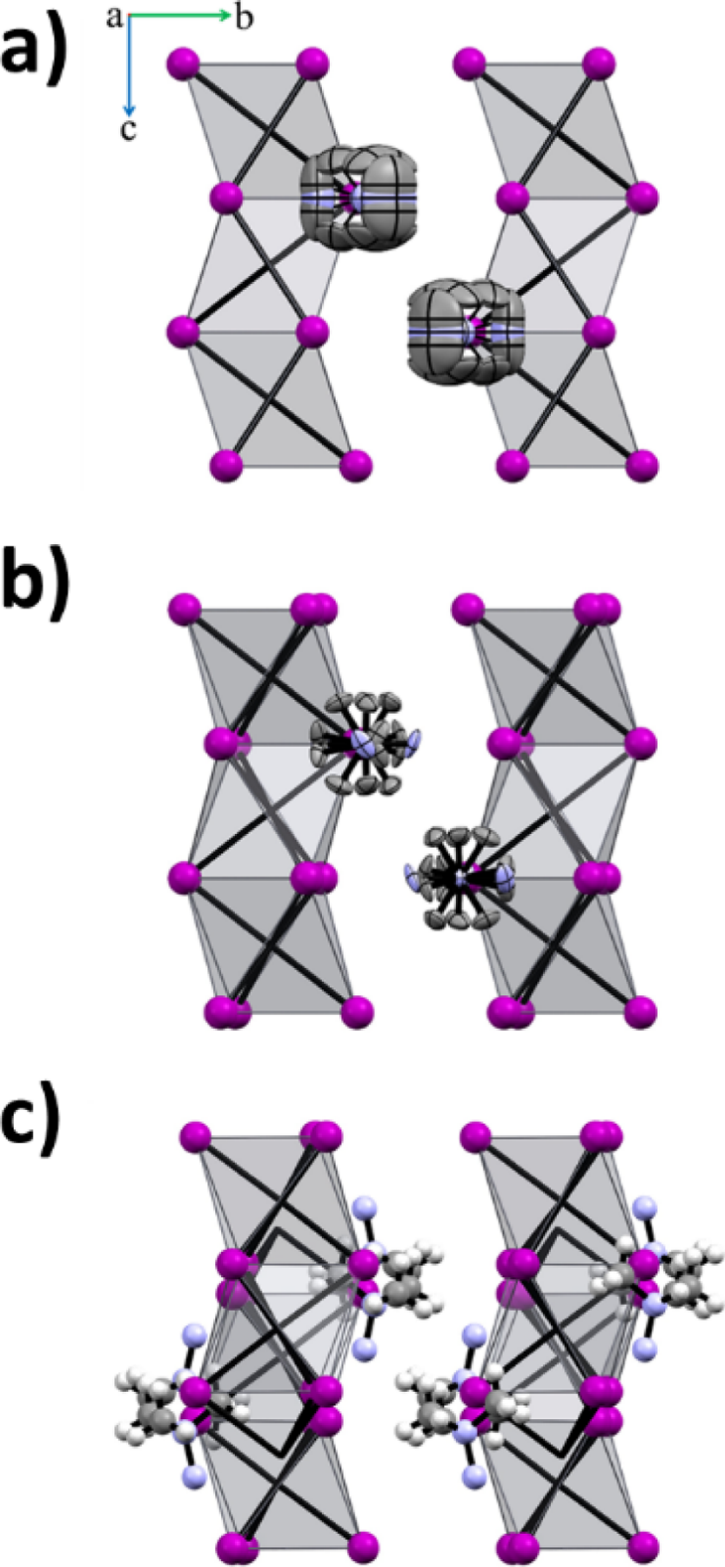
Packing of (Me_3_Hy)[PbI_3_] at 375 K (I) (a) and at 230 K (II) (b) (with the thermal ellipsoids plotted at 30% probability) and at 190 K (III) (c) (structure solved from powder measurement); all structures are plotted along the [100] direction; hydrogen atoms are omitted for clarity.

In phases I and II, the inorganic part of the structure was built by two Pb^2+^ cations and six I^–^ ions. The face-sharing octahedral chain is created by [PbI_6_]^4–^ ions with three iodine atoms shared between every two octahedra (Figure 4).

The polymer chains are oriented along with the [001] direction, and the distance between the Pb atoms in the chain is *c*/2. The distance between adjacent lead atoms in the directions [100] and [010] defines the parameters *a* and *b* of the unit cell and is at 230 and 375 K, respectively, 9.620 and 9.774 Å. The organic part of the unit cell contains Me_3_Hy^+^, located in the voids of the framework. They fill the whole available volume, assuring dense packing. In I and II, cations show significant disorder due to rotation around sixfold inversion axes (N1 nitrogen atom is located at this axis and N2 in the *m* plane). The positions of the organic part are stabilized by a system of N–H···I and C–H···I hydrogen bonds. Due to the high symmetry, the intermolecular interactions are multiplied by symmetry operations, and the robust hydrogen bond network is created. Selected examples of contacts for II were presented in Table S4. For phase I, only C–H···I interactions were found (Table S5). No N–H···I interactions were found for the structure model in the HT phase, which is related to the high measurement temperature resulting in missing hydrogens in the amino group. The hydrogens from the terminal NH_2_ group cannot be observed from electron density because of higher thermal distortion parameters related to the elevated experiment temperature.

**Figure 5:**
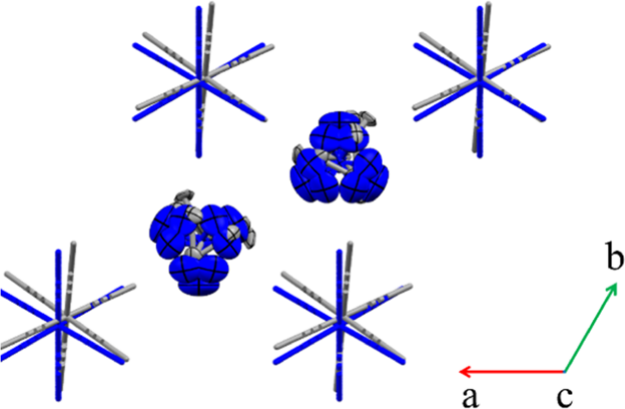
Superposition of (Me_3_Hy)[PbI_3_] structures at 375 K (blue, phase I) and at 230 K (gray, phase II) with the thermal ellipsoids plotted at 30% probability (along the [001] direction); hydrogen atoms are omitted for clarity.

The PT at *T*_1_ is connected with distortions in the organic part. The chain structure is composed of slightly distorted octahedra. It is rigid and remains unchanged with temperature and symmetry increase (Figure S5). The values of the I–Pb–I angles are practically identical for both polymorphs, being ca. 86 and 94°. Similarly, Pb–I distances are nearly identical—3.2361 and 3.2279 Å, in I and II, respectively. It is justified by subtle changes in cell parameters and cell volume. The *c* parameter shows some fluctuations (Figure 5), whereas for *a*, we observed a steady increase. The most linear increase was found for the cell volume, which showed an expansion of ca. 3.6% with a temperature increase from 215 to 375 K.

The single crystal studies for phase III (below 205 K) showed that single crystals crack into several domains related by rotation around the *c* axis (Figure S6). The treatment of them as domains of the twinned crystal failed. These experiments provided cell parameters, but the space group was incorrectly assigned and SHELXT^24^ did not show a reasonable solution in the correct space group. Hence, the powder experiments were carried out (obtained data is in Table S6).

The cell parameters of the LT phase III showed that the volume expanded by approximately four times, and the *c* parameter remained retained. This parameter corresponds to the chain direction, forming the most robust part of the structure. Hence, we can assume that this topology might be preserved. The structure determination from powder data indicated a centrosymmetric *Pbca* space group. The structure clearly confirms that the chain composed of face-sharing PbI_6_ octahedra is robust and did not undergo disruption despite the single crystal integrity being lost. The Pb–I distances range from 3.18 to 3.28 Å (*d*_av_ = 3.244 Å), which is comparable to the phases I and II (*d*_av_ being 3.228 and 3.236 Å for phases II and I, respectively). We conclude that there are subtle changes related to lowered symmetry of the [PbI_6_]^4–^ octahedron (Figure 4 and Table S7). It should be noted that adjacent chains are slightly shifted. It can be assessed by lead atom position—in I and II it is positioned in the (001) plane, whereas in III it is displaced by ca. 0.06 (in fractional coordinates) from this plane. The positions of (Me_3_Hy)^+^ cations in the unit cell show that they were slightly repositioned in the crystal framework (Figure S7), which should affect hydrogen bonds network. Moreover, PT results in reorientation of the cations in phase III, causing that the N–N bonds to be approximately parallel to the chain direction. It is conceivable that this change affects the hydrogen bond network. This effect cannot be fully discussed because in the final model, two hydrogen atoms from the NH_2_ group are missing. Nevertheless, the organic cation is trapped in the crystal network by a robust network of C–H···I hydrogen bonds (Table S8). Summarizing, we can hypothesize that crystal cracking below *T*_2_ and PT are related to the mutual shift of adjacent chains, the relocation of the cation and its reorientation in terms of the N–N bond position according to the chain propagation direction. All these factors can affect the N–H···I and C–H···I hydrogen bond network. These structural changes are much more severe than those between I and II phases and justify the big entropy change as well as distinct features observed in IR and Raman spectra (see Temperature-Dependent Raman and IR Studies).

### Vibrational Properties

#### Molecular Vibrations of the (Me_3_Hy)^+^ Cation

The free Me_3_Hy^+^ cation has 42 vibrational degrees of freedom, including 14 stretching (ν) and 28 deformational modes. The stretching modes can be described as ν_s_NH_2_ (symmetric), ν_as_NH_2_ (antisymmetric), 3× ν_s_CH_3_, 6× ν_as_CH_3_, νNN, ν_s_CNC, and ν_as_CNC, whereas the bending as δNH_2_ (bending), ρNH_2_ (rocking), τNH_2_ (twisting), ωNH_2_ (wagging), 3× δ_s_CH_3_, 6× δ_as_CH_3_, 6× ρCH_3_, 3× τCH_3_, 3× δCNN, and 3× δCNC.

#### Assignment of Bands

RT Raman and IR spectra are presented in Figure S8. The proposed assignment, presented in Table S9, was based on comparative analysis with literature sources, including papers describing IR and Raman spectra of 1,1,1-methylhydrazinium iodide,^35^ coordination nickel(II) compound with 1,1,1-trimethylhydrazinium as ligand^36^ and perovskite-like compounds templated by 1,1-dimethylhydrazinium^22^ and methylhydrazinium^37,38^ cations. Bands corresponding to internal vibrations of Me_3_Hy^+^ are observed in a characteristic ranges as reported in literature.

The strongest Raman band, which has a maximum at 105 cm^–1^ and an IR counterpart at 101 cm^–1^, was assigned to Pb–I stretching modes, while the weaker Raman band, which has a maximum at 60 cm^–1^, was attributed to bending I–Pb–I vibrations as well as librational motions of the entire PbI_6_ units.

#### Temperature-Dependent Raman and IR Studies

In order to understand the mechanisms of PTs, IR and Raman spectra were measured upon heating as a function of temperature in the range of 80–400 K (see Figures S9–S13). As may be seen, IR and Raman spectra evolve with noticeable changes at *T*_2_ and very minor changes at *T*_1_. This is consistent with the XRD measurements, which demonstrate an increase in symmetry from orthorhombic to hexagonal at *T*_2_ and maintain hexagonal symmetry at *T*_1_. Furthermore, it can be noticed that PT at *T*_2_ causes strong splitting of the IR and Raman bands, which are due to symmetry change and the expected increased number of inequivalent Me_3_Hy^+^ cations in phase III. Furthermore, all the IR and Raman bands are very narrow in phase III, suggesting that the organic cations become fully ordered. The abrupt nature of the changes seen at *T*_2_ demonstrates that organic cations completely order shortly below *T*_2_ rather than gradually freeze upon cooling.

**Figure 6:**
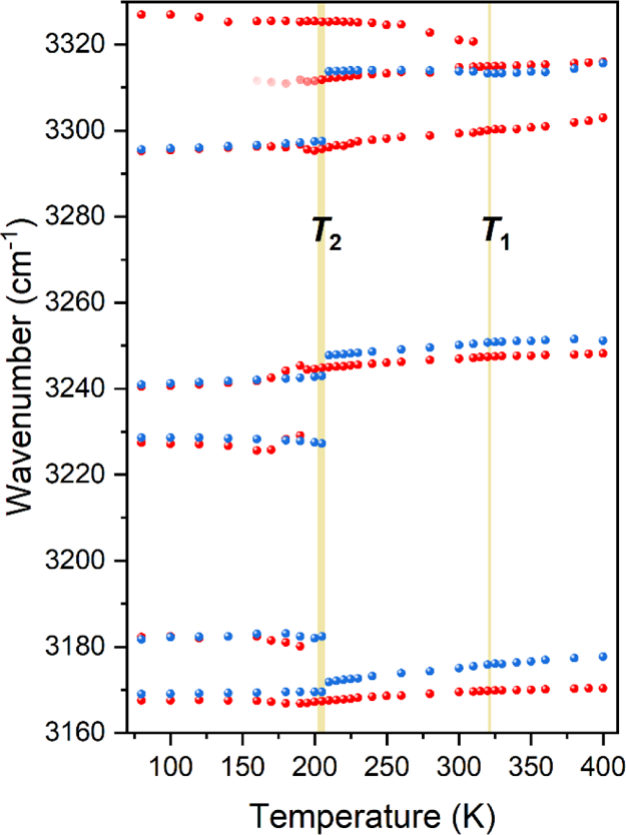
Thermal evolution of positions of the Raman (blue) and IR (red) bands corresponding to stretching vibrations of the amino group; vertical lines correspond to temperatures of PTs determined from DSC.

**Figure 7:**
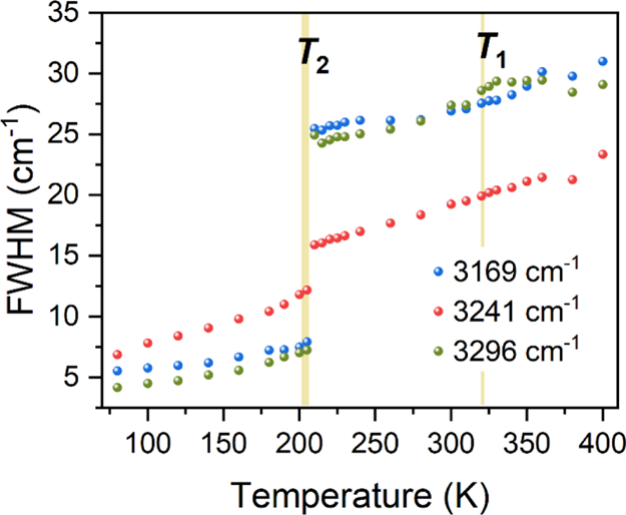
Thermal evolution of fwhms of the Raman bands corresponding to stretching vibrations of the amino group; vertical lines correspond to temperatures of PTs determined from DSC.

The Me_3_Hy^+^ cation is distinct among other hydrazine derivatives since only one amino group may create hydrogen bonding with the PbI_6_ octahedra. The positions of bands corresponding to stretching vibrations of the NH_2_ terminal group are a determinant of the strength of these interactions. Lorentz curves were used to fit spectra in order to identify shifts in band maxima and variations in full-width-at-half-maxima (fwhm) with temperature. Figures 6 and 7 show results obtained for stretching vibrations of the NH_2_ terminal group. The positions of bands and fwhms hardly change during PT at *T*_1_. In contrast, at *T*_2_, a majority of bands are downshifted in phase III. The most pronounced shifts, by 16.4 and 15.8 cm^–1^, were noticed for the Raman band at 3313 cm^–1^ and the IR band at 3314 cm^–1^, respectively (see Figure 6). Additionally, while changes in fwhms at *T*_1_ are undetectable, shifts at *T*_2_ are accompanied by a considerable strong narrowing of bands, decreased most by 17.7 cm^–1^ for the Raman band at 3313 cm^–1^ (Figure 7). These findings suggest that the HB network is irrelevant to the mechanism of PT at *T*_1_, while it is reorganized and strengthened during PT at *T*_2_, which is driven by the ordering of the Me_3_Hy^+^ cations. This implies that phase III is stabilized by stronger HBs, which prevent further disorder of the cations and cause their lock-in. The same results are achieved by observing other characteristic vibrations related to the amino group (Figure S14). In addition, the δNH_2_ and ρNH_2_ vibrations are more sensitive to PT, exhibiting modest inflections at *T*_1_.

The thermal behavior of vibrational modes corresponding to the skeleton of Me_3_Hy^+^ cations (without H atoms) is depicted in Figure S15. Changes at *T*_1_ are, as predicted, within the fitting error, but they are apparent at *T*_2_. However, the changes at *T*_2_ are minor, implying that the geometry of the skeleton is only marginally changed during this PT.

The positions of bands corresponding to stretching and bending vibrations of methyl groups shift during PT at *T*_2_ (see Figure S16), which supports the dynamic character of this PT. The variations at *T*_1_ are less pronounced because they are likely related to confinement changes induced by changes in cell volume.

The significant splitting and narrowing of low-wavenumber Raman modes below *T*_2_ demonstrate that the symmetry is reduced to orthorhombic (Figure S17). The splitting of the Raman band near 100 cm^–1^ into three narrow components also demonstrates the presence of unequal Pb–I bonds in the LT phase. This kind of splitting needs to be connected with distortion of the lead-iodide chains. Moreover, the abrupt evolution of Raman spectra at *T*_2_ confirmed the discontinuous nature of the transformation.

#### High-Pressure Raman Studies

**Figure 8:**
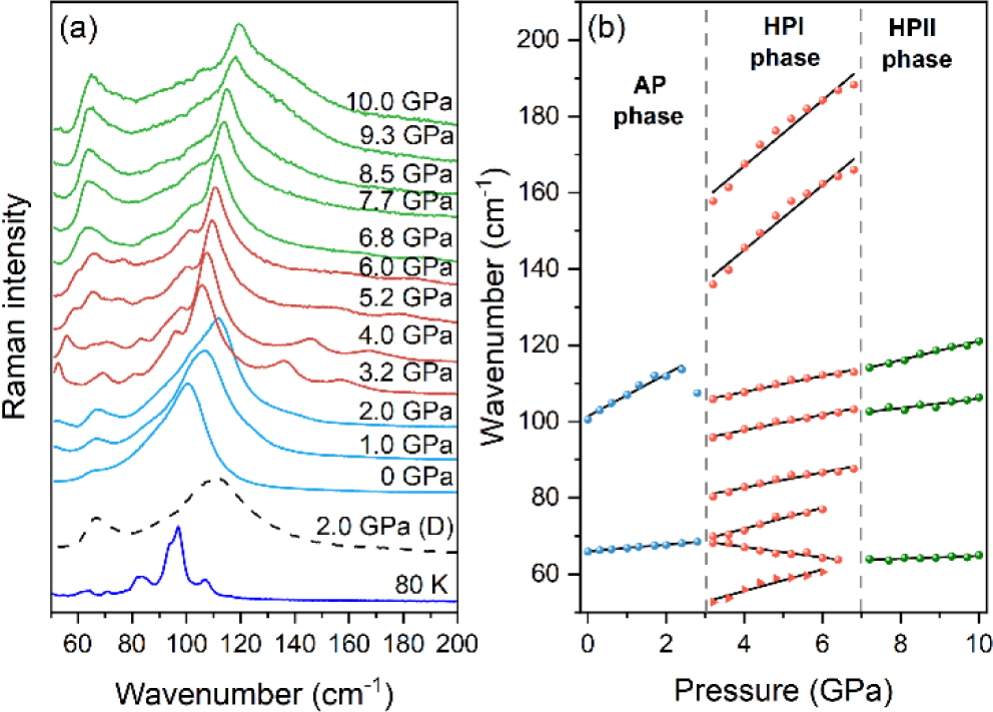
(a) Low-wavenumber Raman bands in the 0–10.0 GPa range during compression compared to a spectrum at 2.0 GPa measured during decompression (D, dashed line) and Raman spectrum obtained at ambient pressure at 80 K; (b) pressure evolution of Raman bands; the vertical dashed lines in (b) indicate the high-pressure PTs.

In order to better understand the stability of (Me_3_Hy)[PbI_3_], the low-wavenumber Raman spectra were measured as a function of pressure (Figure 8a). Figure 8b presents the pressure-dependent evolution for the observed modes. The values of pressure coefficients (α = dω/d*P*) and wavenumber intercepts at zero pressure (ω_0_) derived by fitting the experimental data with a linear function ω(*P*) = ω_0_ + α*P* are shown in Table S10.

At first, the increase in pressure causes only minor changes. At 2.0 GPa, the components of the strongest band at 100 cm^–1^ start to experience intensity changes. At 3.2 GPa, the spectrum changes qualitatively, including the appearance of new bands, indicating a high-pressure PT (1st high-pressure phase, HPI) occurring between 2.8 and 3.2 GPa. The fact that there are more bands suggests that the HPI phase has lower symmetry than ambient-pressure (AP) phase. It is also possible that the symmetry of the HPI phase is orthorhombic based on the comparable number of bands observed at ambient pressure at 80 K (see Figure 8a). Strong narrowing of low-wavenumber modes also implies that the HPI phase may be ordered.

Furthermore, α coefficients change values, which confirms the PT between 2.8 and 3.2 GPa. Bands shift strongly toward higher wavenumber with increasing pressure in both the AP and HPI phases, indicating significant compression of the Pb–I bonds, similar to MHyPbCl_3_.^39^ A few Raman bands disappear when the pressure rises to 6.4–6.8 GPa, and the remaining bands broaden. This may simply be another PT to 2nd HP (HPII) or start of amorphization. The quality of the crystal remains unchanged up to 10 GPa and after decompression (see Figure S18). However, without more investigation, this is difficult to address. The process is entirely reversible, whether or not there is partial amorphization (see Figure 8a).

### Optical Properties

The diffuse reflectance spectrum of (Me_3_Hy)[PbI_3_] crystals recorded at RT is presented in Figure S19a. It consists of a broad band in the 200–500 nm range. On its edge (at 400 nm) one can observe an excitonic band characteristic for halide perovskites.^10,40^ The recalculation of the data obtained from the absorption measurements made it possible to determine the energy band gap of the analyzed compound using the Kubelka–Munk function , where *R* stands for reflectance.^41^ The band gap energy (*E*_g_) of the (Me_3_Hy)[PbI_3_] crystals was calculated to be 3.20 eV (Figure S19b). The obtained value is similar to the size of the energy band gap of other 1D iodide hybrid perovskites (2.58–3.18 eV),^42^ but higher compared to organic or inorganic 3D analogues such as MAPbI_3_ (1.57 eV), FAPbI_3_ (1.48 eV), or CsPbI_3_ (1.73 eV).^43^

**Figure 9:**
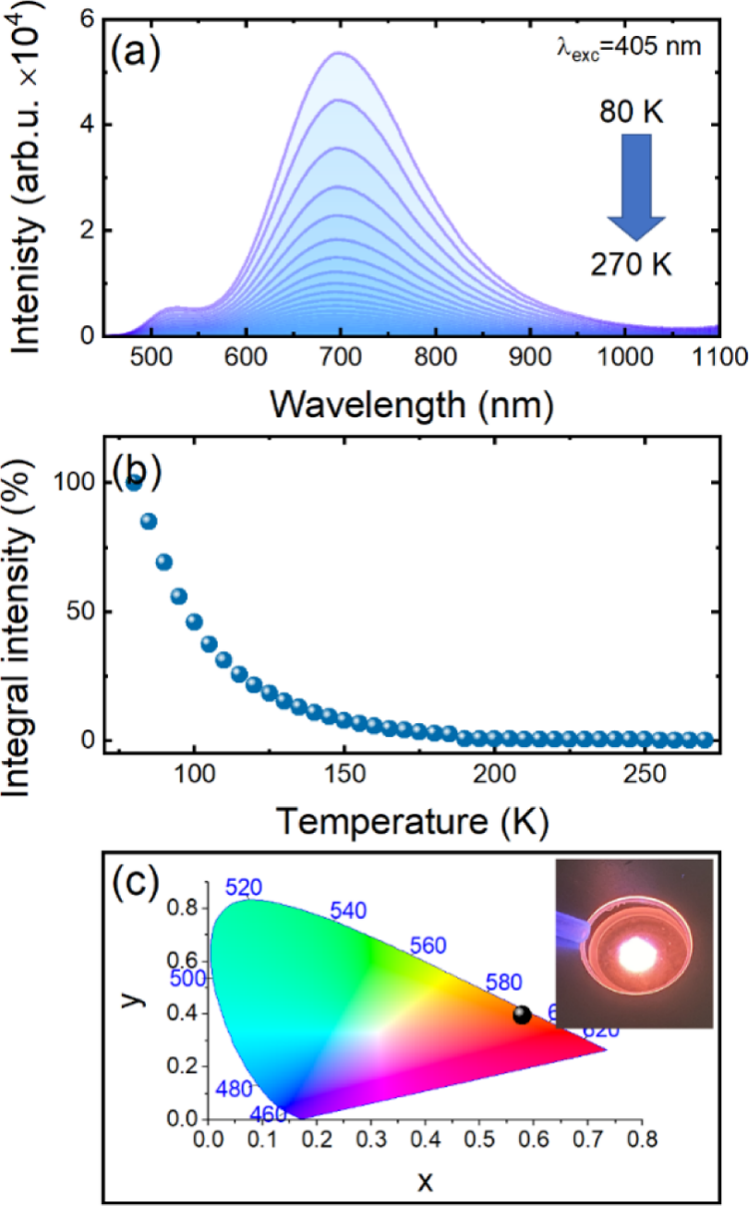
Temperature-dependence emission spectra (a), integral emission intensity (b) and the CIE chromaticity coordinates (c) of (Me_3_Hy)[PbI_3_] crystals. Inset presents the photograph of the glowing sample.

The temperature-dependent emission spectra of (Me_3_Hy)[PbI_3_] crystals measured in the range of 80–270 K in 5 K steps at 405 nm is plotted in Figure 9a. It can be seen that the emission bands cover a wide spectral region and exhibit two maxima, weaker and stronger, located at 520 and 700 nm, respectively. It was found that, the most intense luminescence was obtained at 80 K. Heating the sample causes a gradual decrease of its intensity until complete quenching at 190 K (Figure 9b) due to the thermally activated nonradiative decay channels.^44^ The large Stokes shift between the excitonic absorption and the nearest emission band (120 nm) (Figure S20), the large fwhm of the main emission band (over 200 nm), and the strong temperature quenching of luminescence lead to the conclusion that the origin of the transition recorded in Figure 9a is associated with self-trapped excitons (STEs).^10,44−47^ The presence of two bands indicates the existence of two kinds of defects with different depths. It is worth noting that excitation of the sample with the 405 nm line or with laser diodes of higher energy, that is, 266 and 375 nm, results in the absence of an emission band that could be assigned to FE (free exciton) and BE (bounded exciton), as shown in Figure S21. Since the position of the emission bands does not change with increasing temperature, the chromaticity coordinates were determined for the measurement performed at 80 K and presented on the CIE diagram (Figure 9c).

In order to study the thermal kinetics of (Me_3_Hy)[PbI_3_] crystals, the following expression was used: , where *I* is the intensity, *I*_0_ is the initial intensity at LT, Γ_v_ denotes the radiative decay rate, Γ_0_ is the attempt rate for thermal quenching, and Δ*E* is the activation energy for thermal quenching.^48^ This equation has been recalculated as , in order to determine the activation energy of the investigated material (Δ*E* is the slope of  as a function of 1/*kT* and  is a constant). The calculated activation energy of (Me_3_Hy)[PbI_3_] crystals was determined to be 65 meV (Figure S22).^49^

The luminescence decay profiles of (Me_3_Hy)[PbI_3_] crystals recorded at the 405 nm excitation line, monitored at 520 and 700 nm at 80 K, corresponding to the two peaks observed in the emission spectrum, are presented in Figure S23. It was found that analyzed compound exhibits curves that can be fitted by a double exponential function regardless of the monitored wavelength using the following expression , where *I*(*t*) is the emission intensity, *A*_1_ and *A*_2_ are fitting constants, and τ_1_ and τ_2_ are their corresponding lifetimes. The lifetimes determined for both emission peaks observed at 520 and 700 nm are τ_1_ ≈ 260 ns and τ_2_ ≈ 725 ns. Two components visible in the luminescence decays indicate the existence of non-radiation energy transfers in the (Me_3_Hy)[PbI_3_] crystals. The obtained values are very similar for both bands, which proves the same nature of the defects.

## Conclusions

We synthesized a new perovskitoid 1,1,1-trimethylhydrazinium lead iodide, composed of 1D face-shared chains of [PbI_6_]^4–^ octahedra along the [001] direction and disordered organic cations, to evaluate its structural, thermal, phonon, and emission properties using a variety of techniques. DSC measurement revealed the presence of two PTs at 322 (320) K and 207 (202) K upon cooling (heating), and the TGA measurement demonstrated that the material is stable up to 300 °C.

DSC, single-crystal XRD, and vibrational studies revealed that the transformation from the RT *P*6_3_/*m* phase to the HT *P*6_3_/*mmc* phase has a second-order nature and is associated with minor structural changes. In contrast, the PT to the LT orthorhombic (*Pbca*) phase has a first-order nature and is characterized by a significant change in entropy. It is associated with the ordering (locking) of the organic cations, rearrangement and strengthening of hydrogen bonds, deformation of the lead-iodide octahedral chains, and an increase in the number of inequivalent structural units. The abrupt character of this PT displays the desired switchable dielectric behavior between low (off) and high (on) dielectric states, designating it as a potentially switchable material.

The high-pressure Raman data indicated the presence of two high-pressure PTs, in the 2.8–3.2 and 6.4–6.8 GPa ranges. The low-pressure PT is associated with the ordering of the organic cations, compression of PbI_6_ octahedra, and distortion of the lead-iodide framework, while the second PT is most likely related to the partial and reversible amorphization.

The band gap energy of the crystals, as determined by optical measurements, is 3.20 eV. At LTs, they emit a reddish-orange broad-band emission that is related to STEs. The activation energy of the emission was found to be 65 meV.

## Supporting Information Available

Powder XRD, DSC trace, frequency dependencies of the complex dielectric permittivity, complex electrical modules, packing diagrams for phases I, II, and III, RT and temperature-dependent Raman and IR spectra, IR spectrum, positions and bandwidths of selected IR and Raman bands as a function of temperature, pictures of crystals during HP Raman experiment, diffuse reflectance spectrum, emission spectra measured under 266 and 375 nm excitation, crystal data and structure refinement for I and II phases, selected geometric parameters for phases I and II, assignment of IR and Raman bands, Raman pressure intercepts and pressure coefficients (PDF), and crystal structure of (Me_3_Hy)[PbI_3_] at 230 K (CIF) and 375 K (CIF) (PDF)
