# Supplementary material for: Luminescence and Dielectric Switchable Properties of a 1D (1,1,1-Trimethylhydrazinium)PbI3 Hybrid Perovskitoid
Source: Inorg Chem. 2022 Dec 15;61(51):20886–95. doi: 10.1021/acs.inorgchem.2c03287 (PMC9795545; doi:10.1021/acs.inorgchem.2c03287)
Supplement: Supplementary file 1 — ic2c03287_si_001.pdf [file ic2c03287_si_001.pdf]

Electronic Supplementary Information for

Luminescence and dielectric switchable properties of 1D (1,1,1-trimethylhydrazinium)PbI_3 hybrid perovskitoid

Jan. A. Zienkiewicz,[*,†] Karolina Kałduńska,[§] Katarzyna Fedoruk,[‡] Antonio J. Barros dos Santos[∂], Mariusz Stefanski,[†] Waldeci Paraguassu,[∂] Tadeusz M. Muzioł,[‖] and Maciej Ptak[†]

[†]Institute of Low Temperature and Structure Research, Polish Academy of Sciences, Okólna 2, 50-422 Wrocław, Poland

[‡]Institute of Physics, Wrocław University of Science and Technology, Wybrzeże Wyspiańskiego 27, 50-370 Wrocław, Poland

[§]Department of Biomedical and Polymer Chemistry, Faculty of Chemistry, Nicolaus Copernicus University in Toruń, Gagarina 7, 87-100 Toruń, Poland

[∂]Department of Physics, Federal University of Pará, Campus do Guamá, Rua Augusto Corrêa 01, 66075110 Belém, PA, Brazil

[‖]Department of Inorganic and Coordination Chemistry, Faculty of Chemistry, Nicolaus Copernicus University in Toruń, Gagarina 7, 87-100 Toruń, Poland

Figures

Fig. S1	The comparison of an experimental room-temperature XRD pattern with a simulated one based on the single-crystal measurement at 230 K	S3
Fig. S2	DSC trace for $(Me_3Hy)[PbI_3]$	S4
Fig. S3	TGA curve for $(Me_3Hy)[PbI_3]$	S5
Fig. S4	Frequency dependencies of the complex dielectric permittivity and the complex electrical modules	S6
Fig. S5	Packing of (Me3Hy)[PbI3] at 375 K (phase I) (a) and at 230 K (phase II) (b) (with the thermal ellipsoids plotted at 30% prob-ability) and 190 K (III) (c) (structure solved from powder measurement); all structures are plotted along the [001] direction; hydrogen atoms are omitted for clarity	S7
Fig. S6	Ewald sphere for six domains detected in the cracked single crystal related by rotation around *c* axis	S8
Fig. S7	Packing along the *c* axis in phase III	S9
Fig. S8	Raman (a) and IR (b) spectrum of $(Me_3Hy)[PbI_3]$	S10
Fig. S9	Temperature-dependent Raman spectra in the 3350-2700 cm^{-1} range	S11
Fig. S10	Temperature-dependent Raman spectra in the 1750-150 cm^{-1} range	S12
Fig. S11	Temperature-dependent Raman spectra in the 150-20 cm^{-1} range	S13
Fig. S12	Temperature-dependent IR spectra in the 3400-2750 cm^{-1} range	S14
Fig. S13	Temperature-dependent IR spectra in the 1750-600 cm^{-1} range	S15
Fig. S14	Thermal evolution of Raman (blue) and IR (red) band positions (a), as well as FWHMs of two Raman bands (b) corresponding to bending (δ) and rocking (ρ) vibrations of amino group; vertical lines correspond to temperatures of PTs determined from DSC	S16
Fig. S15	Thermal evolution of positions of the Raman (blue) and IR (red) bands stretching vibrations of CN and NN bonds; vertical lines correspond to temperatures of PTs determined from DSC	S17
Fig. S16	Thermal evolution of Raman (a) and IR (b) band positions corresponding to stretching (a) and bending (b) vibrations of methyl groups, and FWHMs of selected Raman and IR bands (c); vertical lines correspond temperatures of PTs determined from DSC.	S18
Fig. S17	Thermal evolution of low-wavenumber Raman bands	S19
Fig. S18	Changes of $(Me_3Hy)[PbI_3]$ crystal (C) loaded into a 100 μm hole in the stainless steel gasket, along with piece of ruby (R) at selected pressures during compression and after decompression to 4.3 GPa.	S20
Fig. S19	The diffuse reflectance spectrum of $(Me_3Hy)[PbI_3]$ crystals (a) and the calculation of its energy band gap by the Kubelka-Munk function (b)	S21
Fig. S20	Diffuse reflectance at 300 K and emission spectra at 80 K of $(Me_3Hy)[PbI_3]$ crystals	S22
Fig. S21	The emission spectra of $(Me_3Hy)[PbI_3]$ crystals measured under 266 (a) and 375 nm (b) excitation	S23
Fig. S22	The activation energy of the thermal quenching of emission bands of $(Me_3Hy)[PbI_3]$	S24

Fig. S23 The luminescent decay profiles of $(Me_3Hy)[PbI_3]$ crystals measured at 80 K S25

Tables

Tab. S1 Crystal data and structure refinement for I and II phases of $(Me_3Hy)[PbI_3]$ S26
Tab. S2 Selected geometric parameters of phase I S27
Tab. S3 Selected geometric parameters of phase II S28
Tab. S4 Bond lengths (Å) of selected intermolecular contacts for phase II (RT) of $(Me_3Hy)[PbI_3]$ S29
Tab. S5 Bond lengths (Å) of selected intermolecular contacts for phase I (HT) of $(Me_3Hy)[PbI_3]$ S30
Tab. S6 Crystal data obtained from the powder XRD experiment for phase III (LT) of $(Me_3Hy)[PbI_3]$ S31
Tab. S7 Geometric parameters (bond lengths [Å] and angles [°]) for phase III (LT) of $(Me_3Hy)[PbI_3]$ S32
Tab. S8 Bond lengths (Å) of selected intermolecular contacts for phase III (LT) of $(Me_3Hy)[PbI_3]$ S33
Tab. S9 Assignment of IR and Raman bands observed for $(Me_3Hy)[PbI_3]$ S34
Tab. S10 Raman pressure intercepts (ω_0) and coefficients (α) for three phases of $(Me_3Hy)[PbI_3]$ together with proposed assignment S35

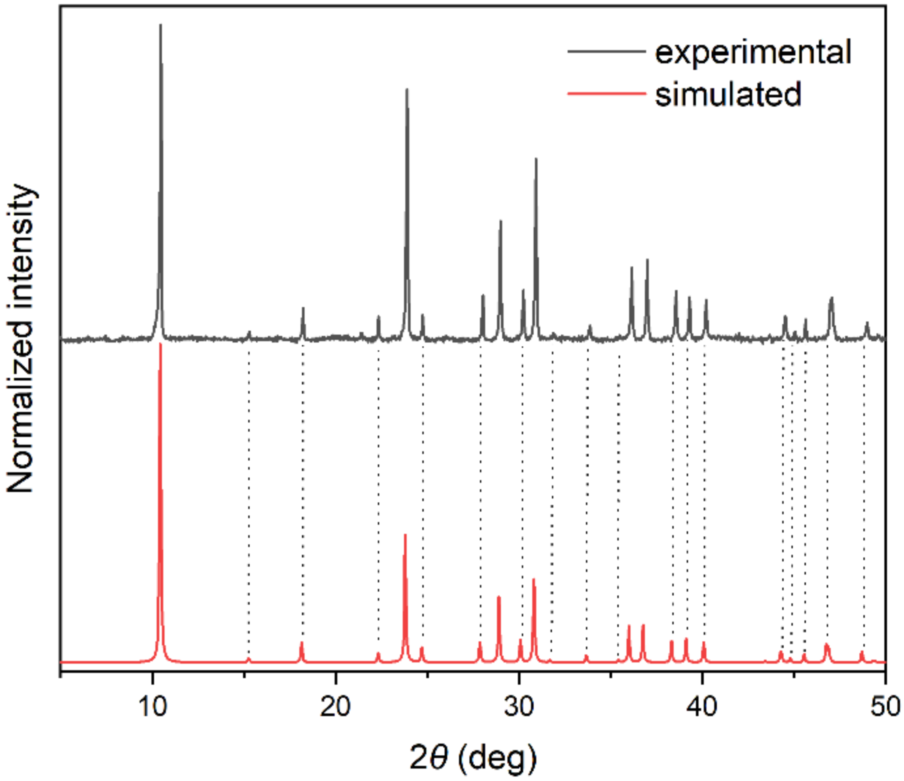

Figure S1. The comparison of an experimental room-temperature XRD pattern with a simulated one based on the single-crystal measurement at 230 K

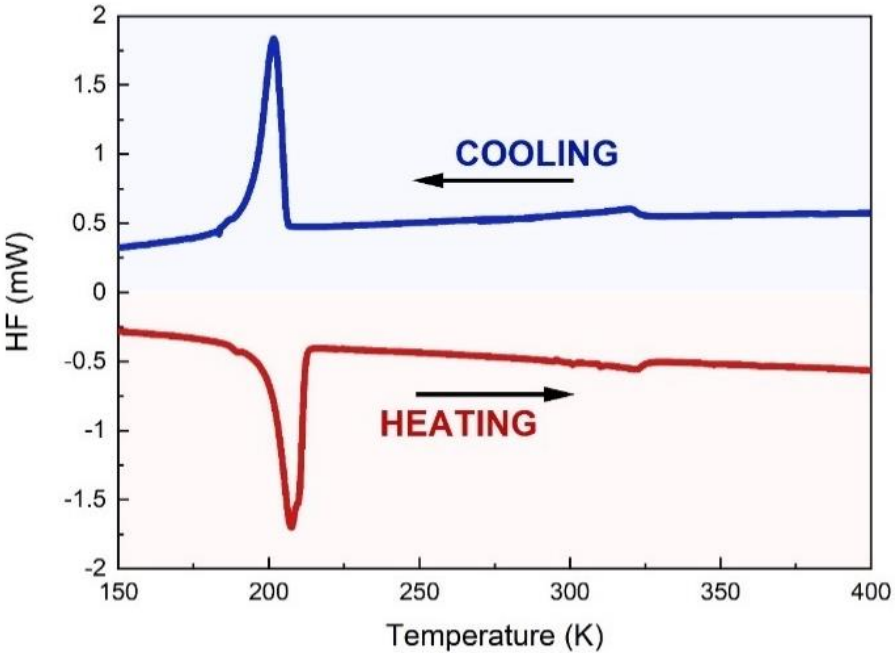

Figure S2. DSC trace for $(Me_3Hy)[PbI_3]$

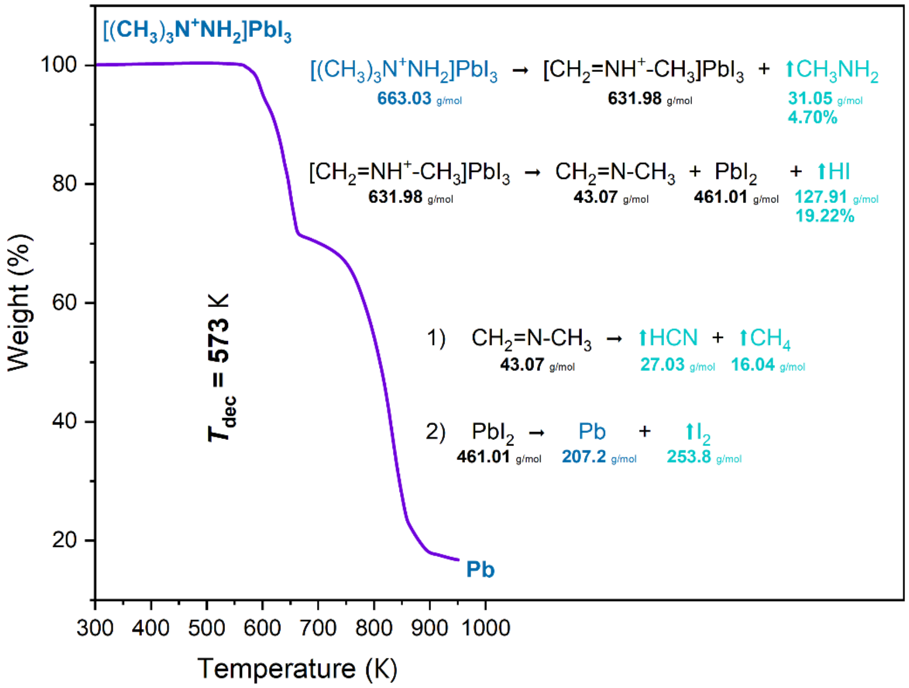

Figure S3. TGA curve for $(Me_3Hy)[PbI_3]$

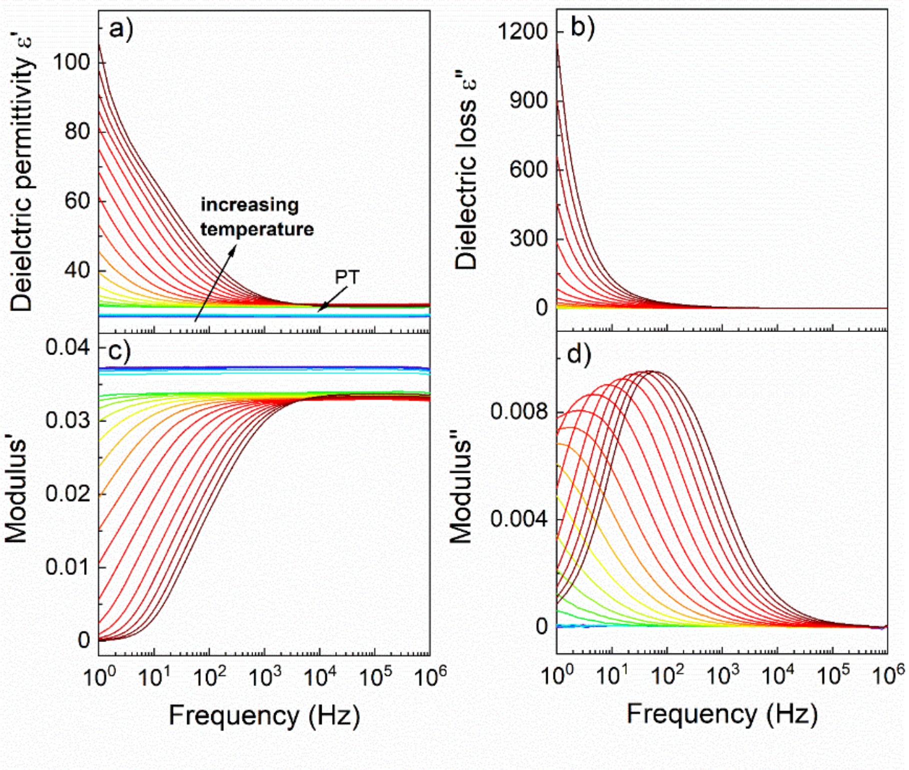

Figure S4. Frequency dependencies of the complex dielectric permittivity and the complex electrical modules

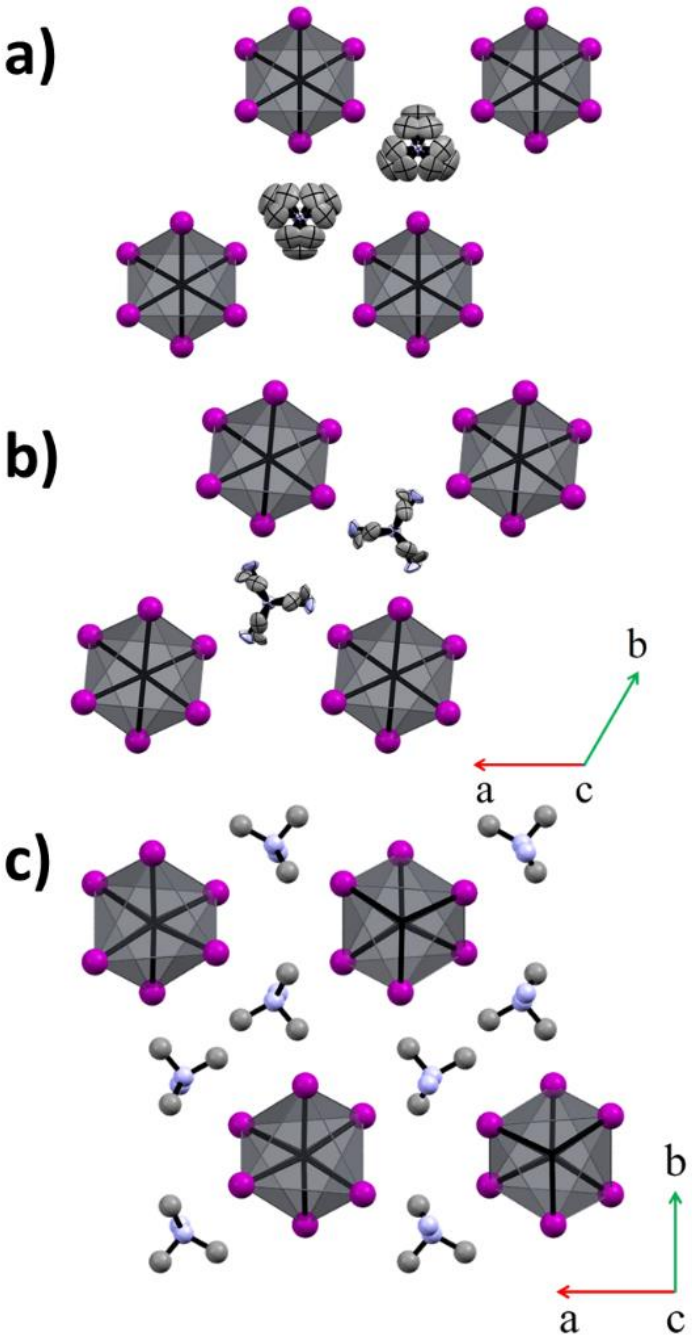

Figure S5. Packing of $(Me_3Hy)[PbI_3]$ at 375 K (phase I) (a) and at 230 K (phase II) (b) (with the thermal ellipsoids plotted at 30% probability) and 190 K (III) (c) (structure solved from powder measurement); all structures are plotted along the [001] direction; hydrogen atoms are omitted for clarity

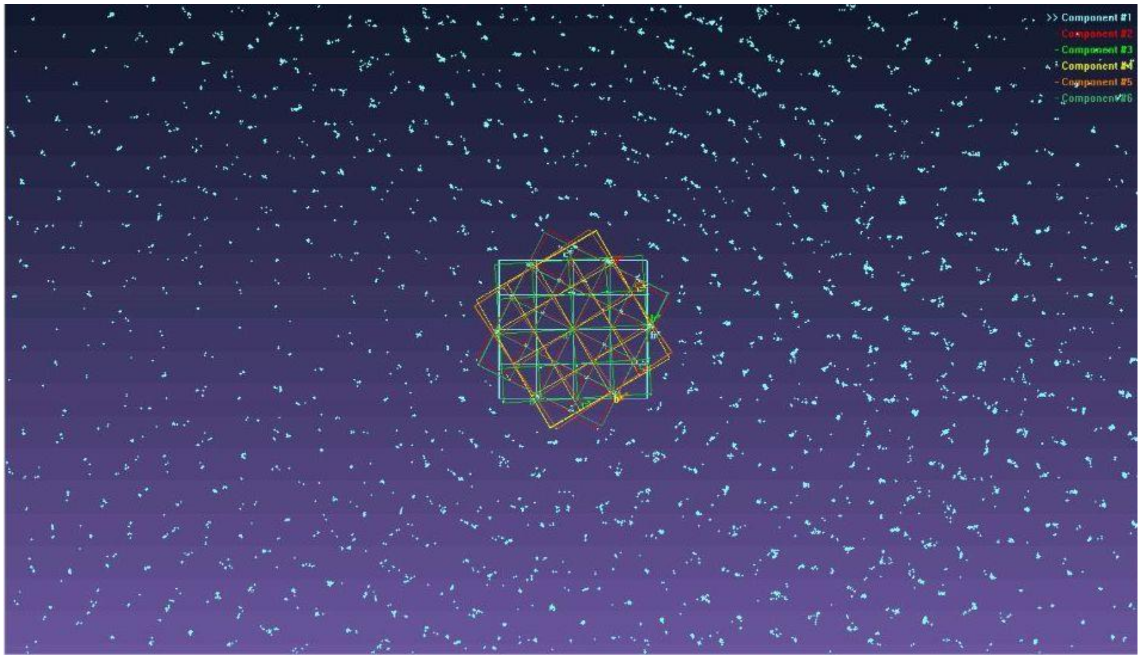

Figure S6. Ewald sphere for six domains detected in the cracked single crystal related by rotation around *c* axis. Their abundance is similar.

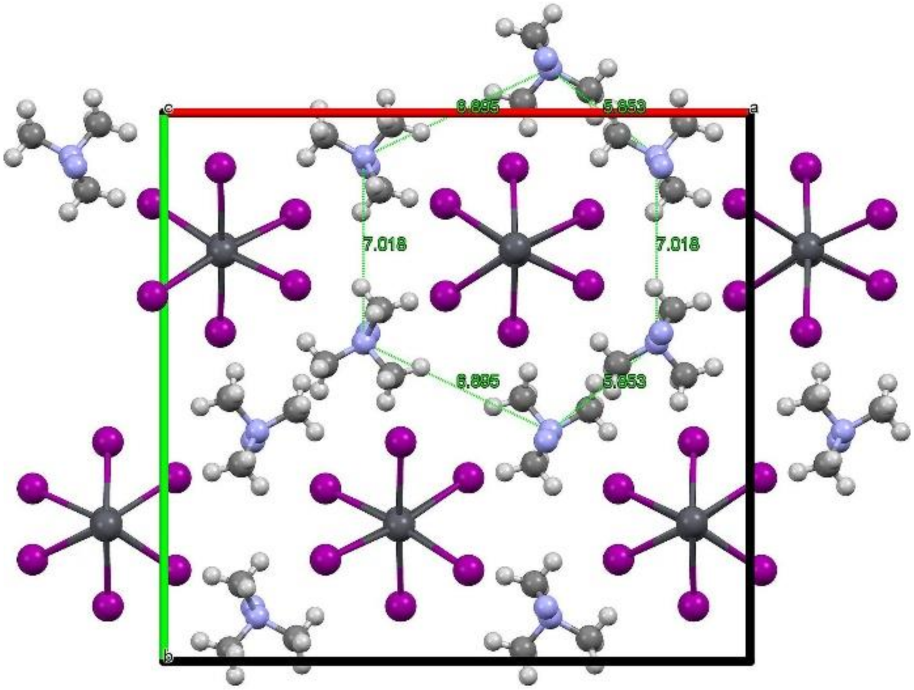

Figure S7. Packing along *c* axis in phase III. It shows maintained chain topology and altered positions of organic cations. Distances are given between central nitrogen atoms of the Me_3Hy^+ cations. Reorientation of the organic cations in terms of N-N bond direction according to chain propagation direction is also visible. In the final model two nitrogen atoms from NH_2 group are missing

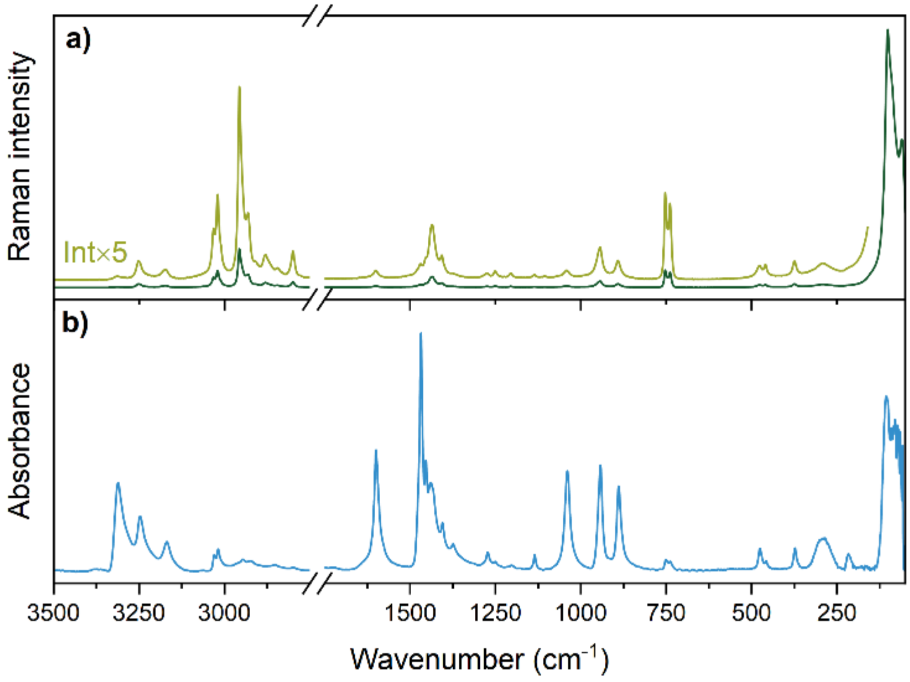

Figure S8. RT Raman (a) and IR (b) spectrum of $(Me_3Hy)[PbI_3]$

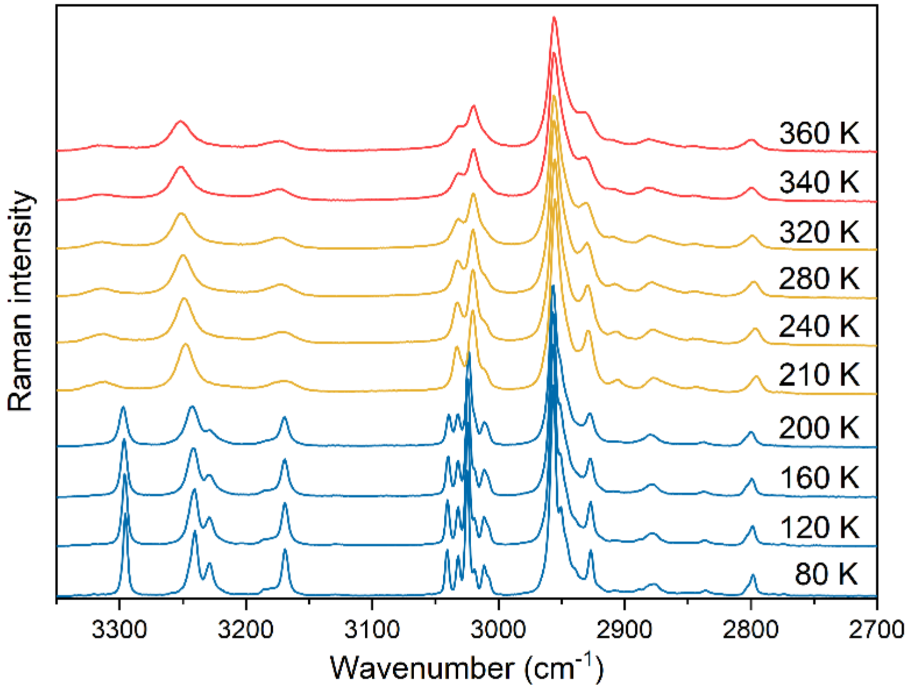

Figure S9. Temperature-dependent Raman spectra in the 3350-2700 cm^{-1} range

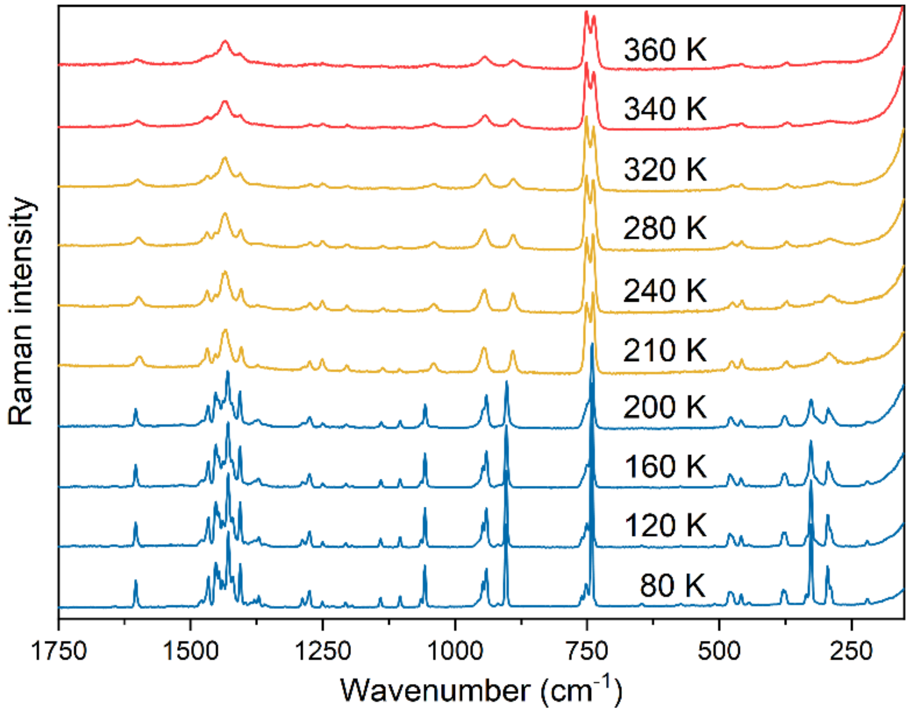

Figure S10. Temperature-dependent Raman spectra in the 1750-150 cm^{-1} range

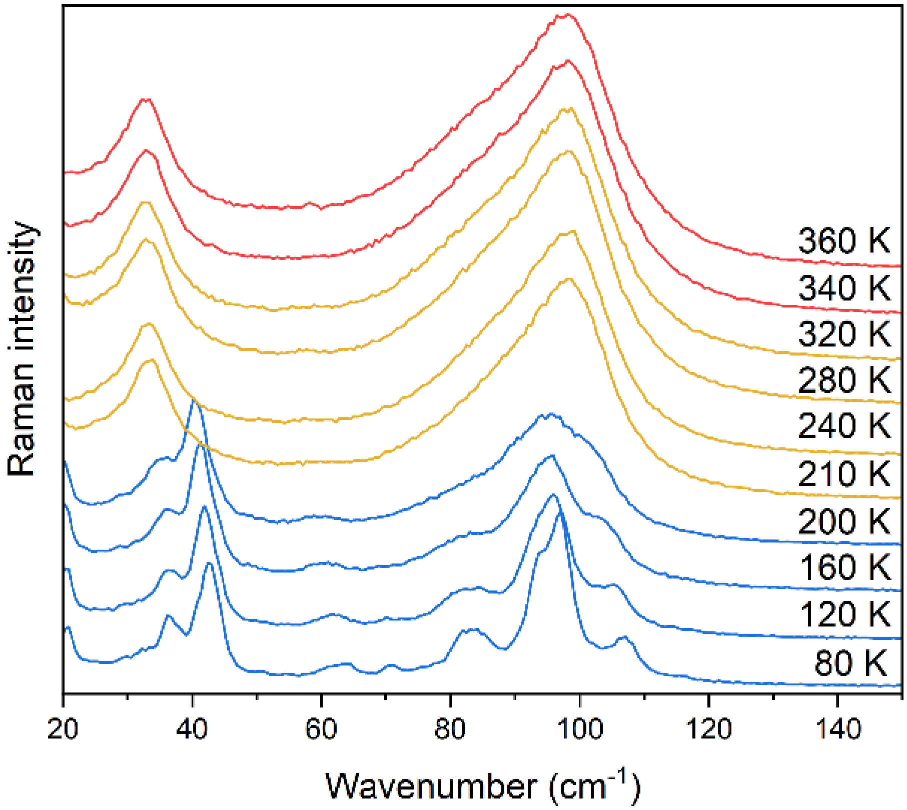

Figure S11. Temperature-dependent Raman spectra in the 150-20 cm^{-1} range

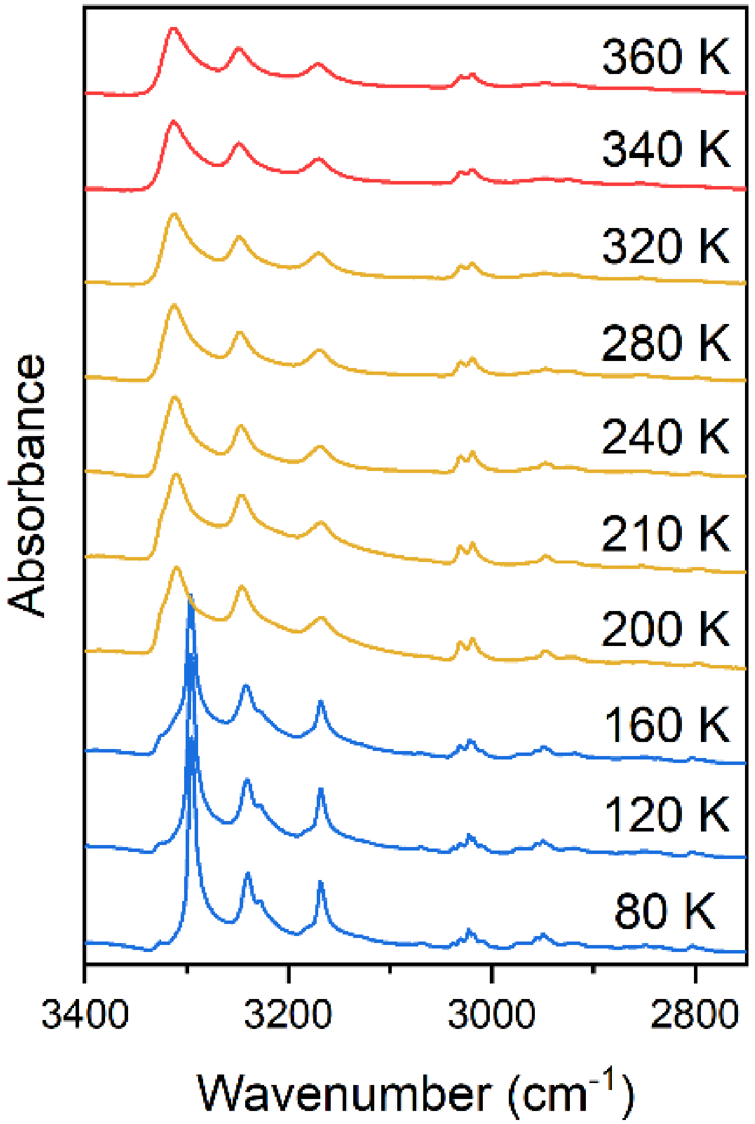

Figure S12. Temperature-dependent IR spectra in the 3400-2750 cm-[1] range

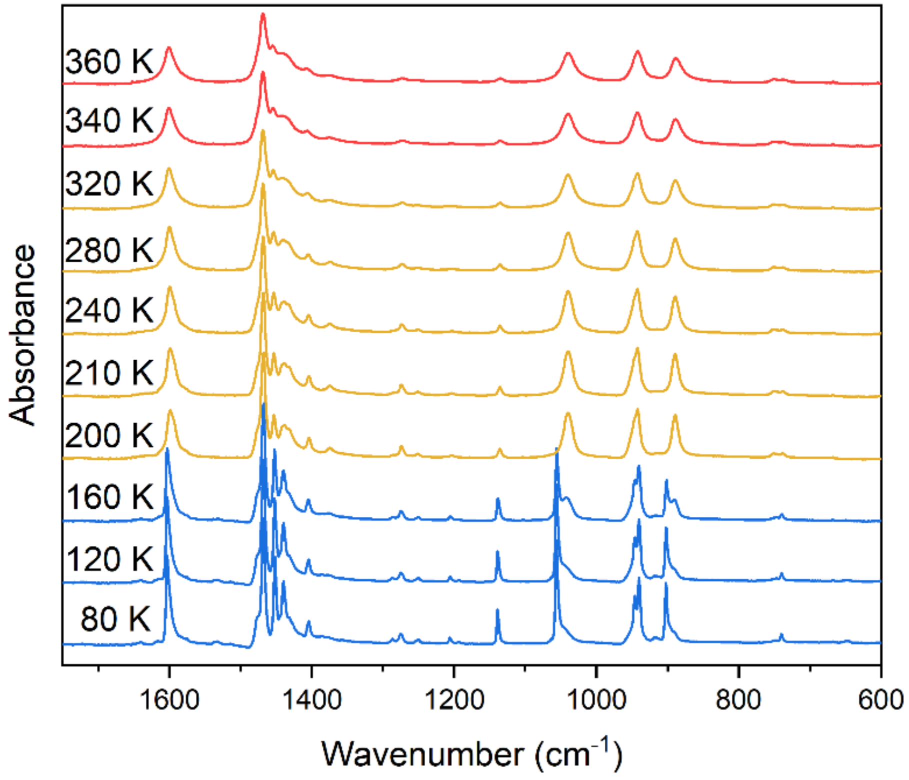

Figure S13. Temperature-dependent IR spectra in the 1750-600 cm-[1] range

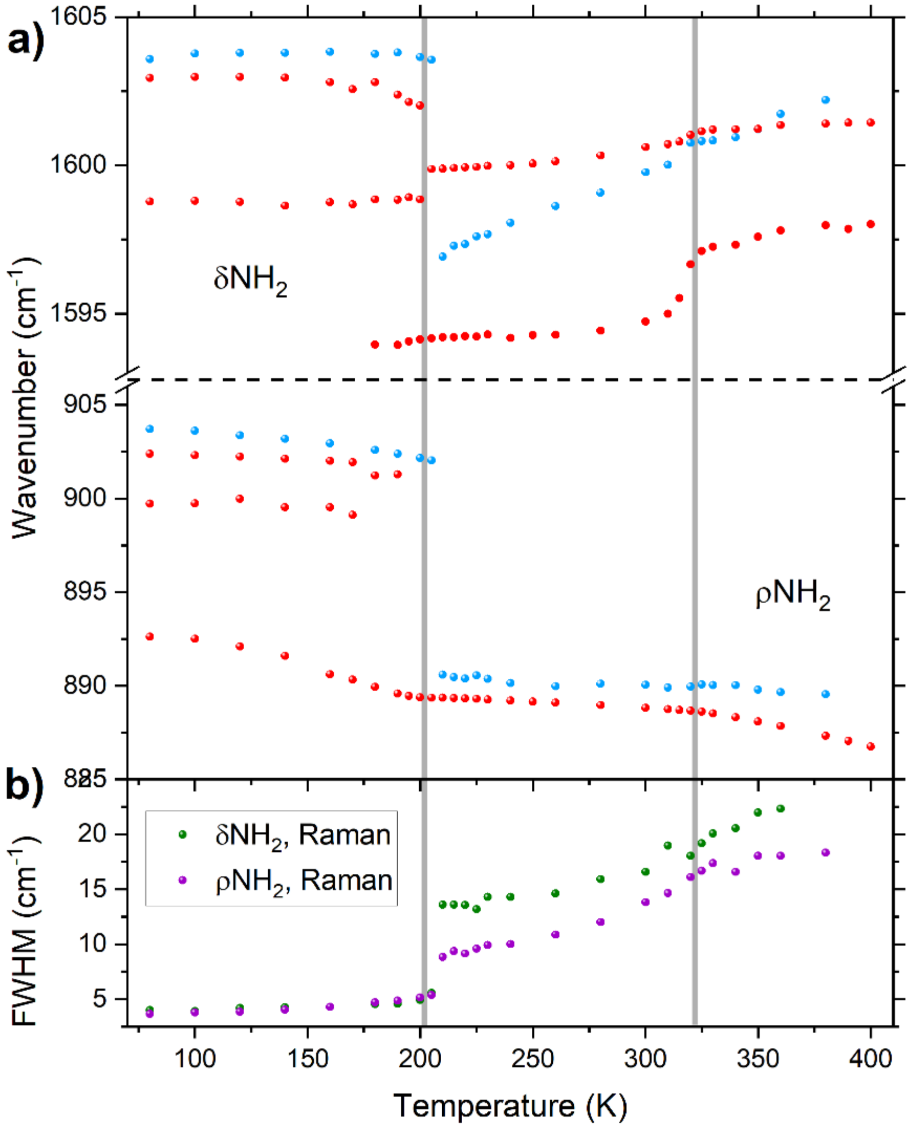

Figure S14. Thermal evolution of Raman (blue) and IR (red) band positions (a), as well as FWHMs of two Raman bands (b) corresponding to bending (δ) and rocking (ρ) vibrations of amino group; vertical lines correspond to temperatures of PTs determined from DSC

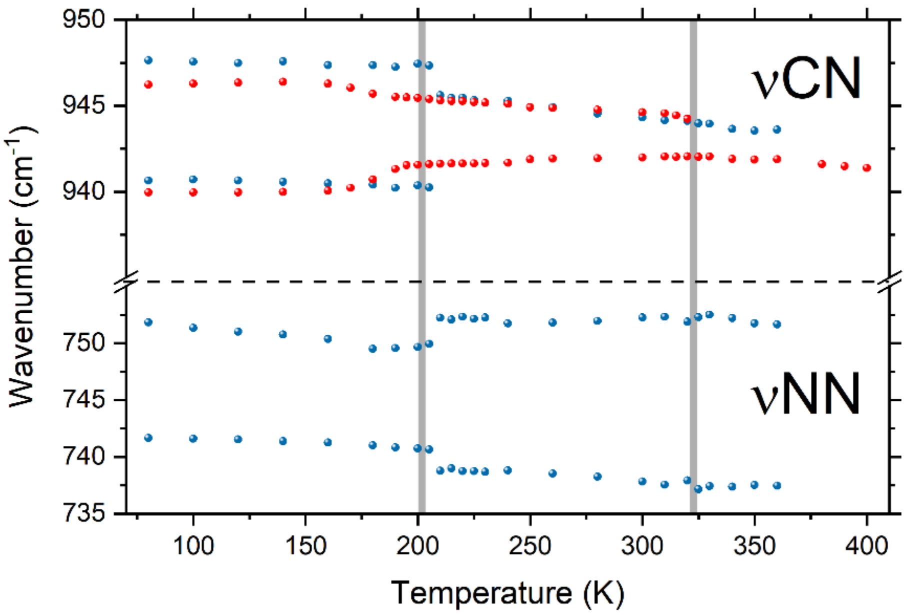

Figure S15. Thermal evolution of positions of the Raman (blue) and IR (red) bands stretching vibrations of CN and NN bonds; vertical lines correspond to temperatures of PTs determined from DSC

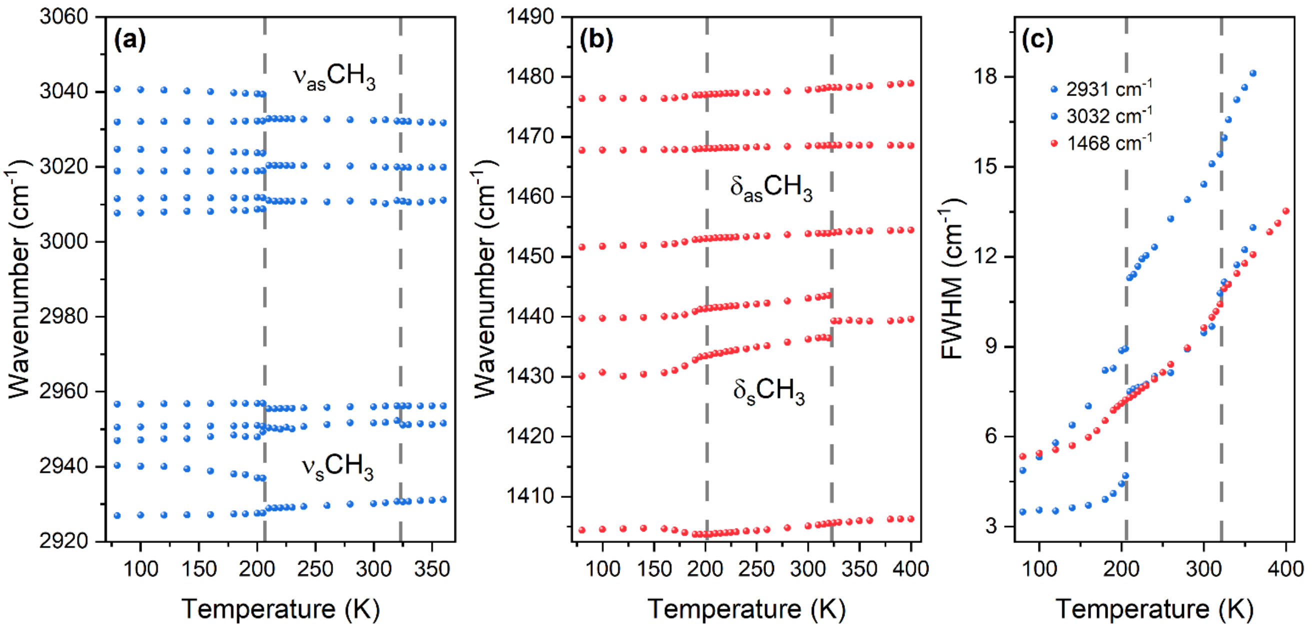

Figure S16. Thermal evolution of Raman (a) and IR (b) band positions corresponding to stretching (a) and bending (b) vibrations of methyl groups, and FWHMs of selected Raman and IR bands (c); vertical lines correspond temperatures of PTs determined from DSC.

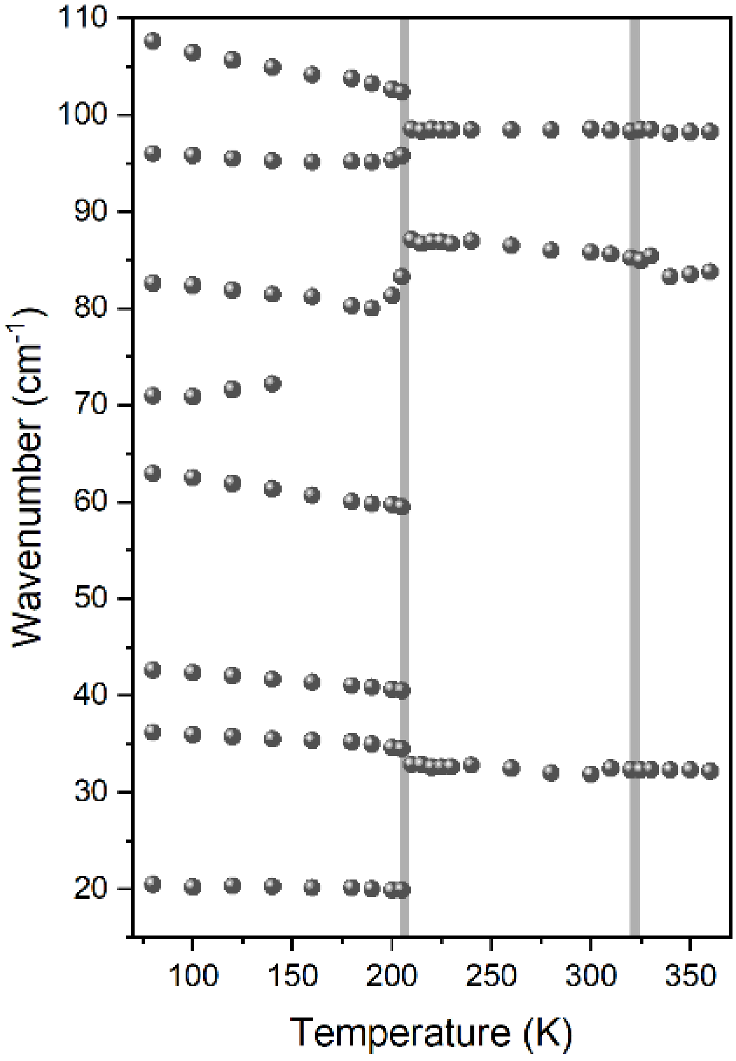

Figure S17. Thermal evolution of low-wavenumber Raman bands

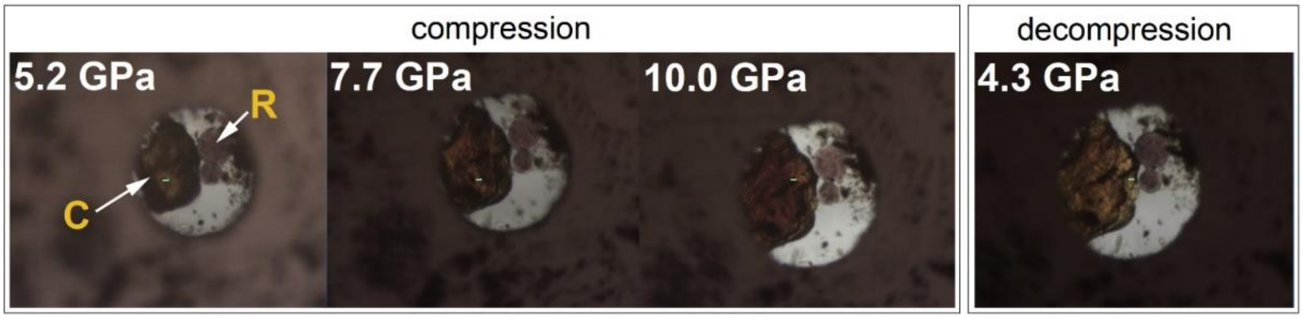

Figure S18. Changes of $(Me_3Hy)[PbI_3]$ crystal (C) loaded into a 100 µm hole in the stainless steel gasket, along with piece of ruby (R) at selected pressures during compression and after decompression to 4.3 GPa.

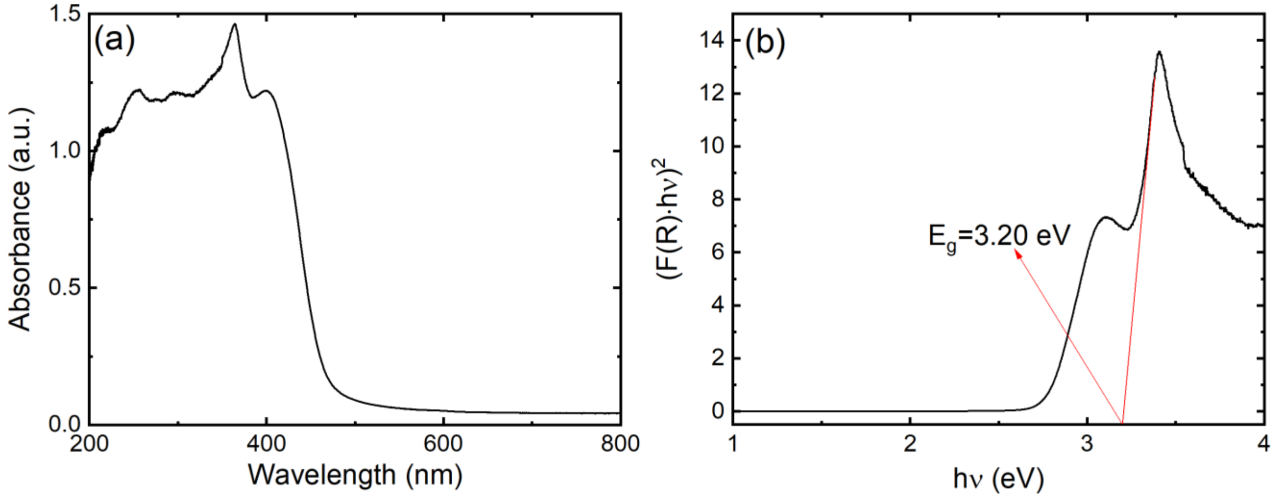

Figure S19. The diffuse reflectance spectrum of $(Me_3Hy)[PbI_3]$ crystals (a) and the calculation of its energy band gap by the Kubelka-Munk function (b)

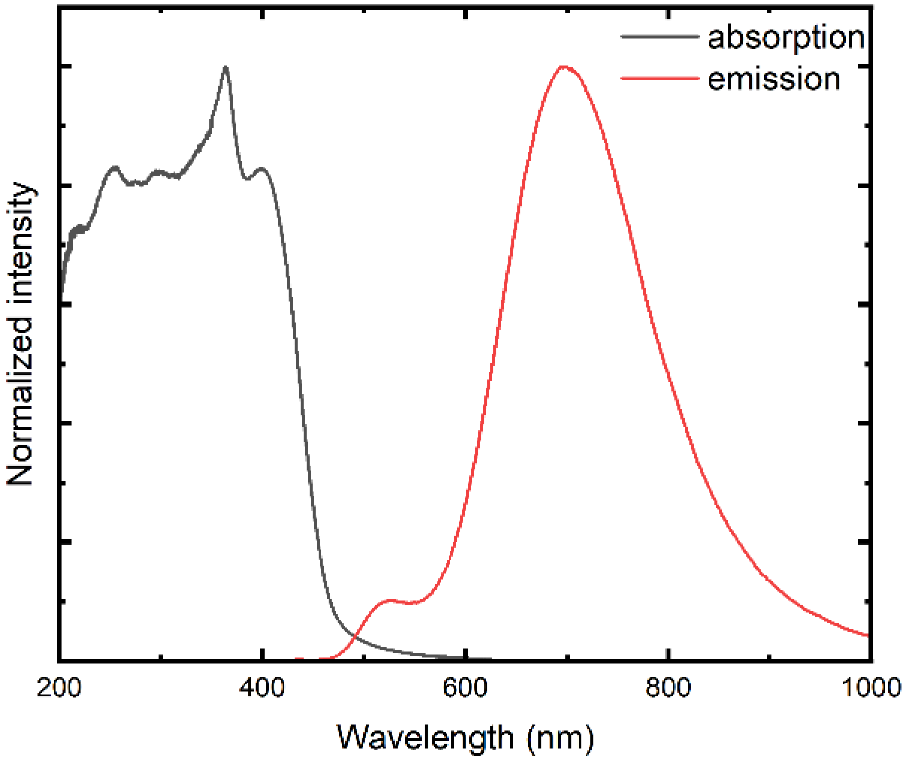

Figure S20. Diffuse reflectance at 300 K and emission spectra at 80 K of $(Me_3Hy)[PbI_3]$ crystals

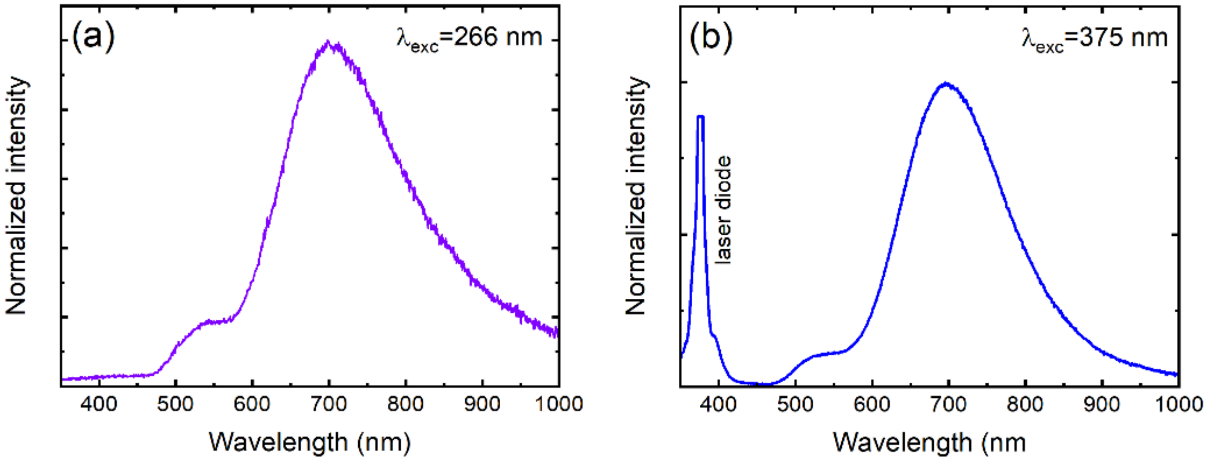

Figure S21. The emission spectra of $(Me_3Hy)[PbI_3]$ crystals measured under 266 (a) and 375 nm (b) excitation

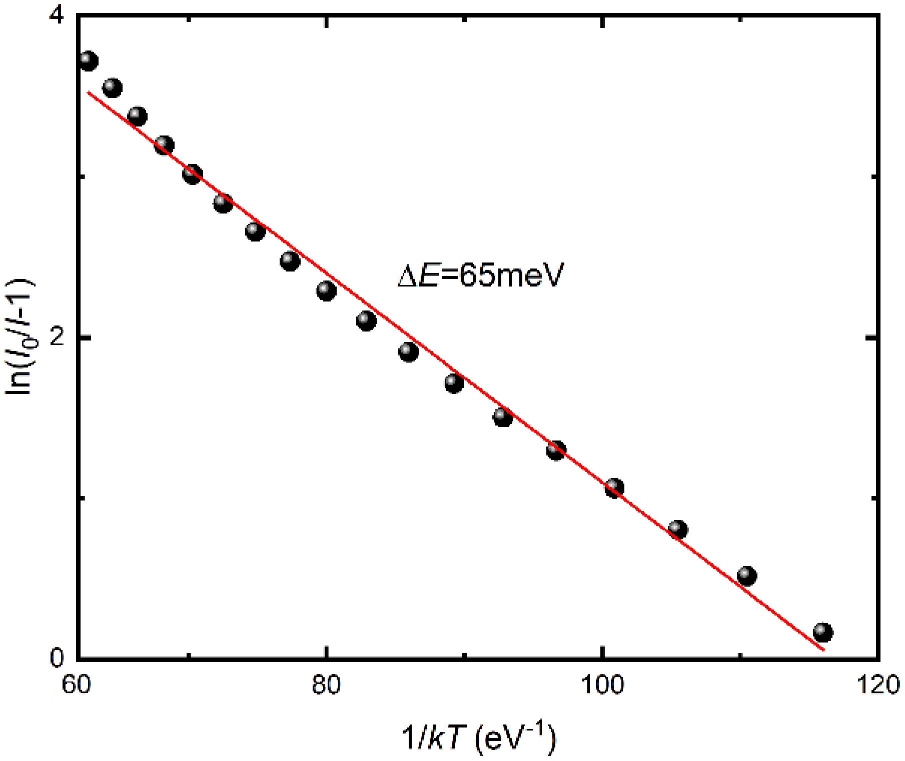

Figure S22. The activation energy of the thermal quenching of emission bands of $(Me_3Hy)[PbI_3]$

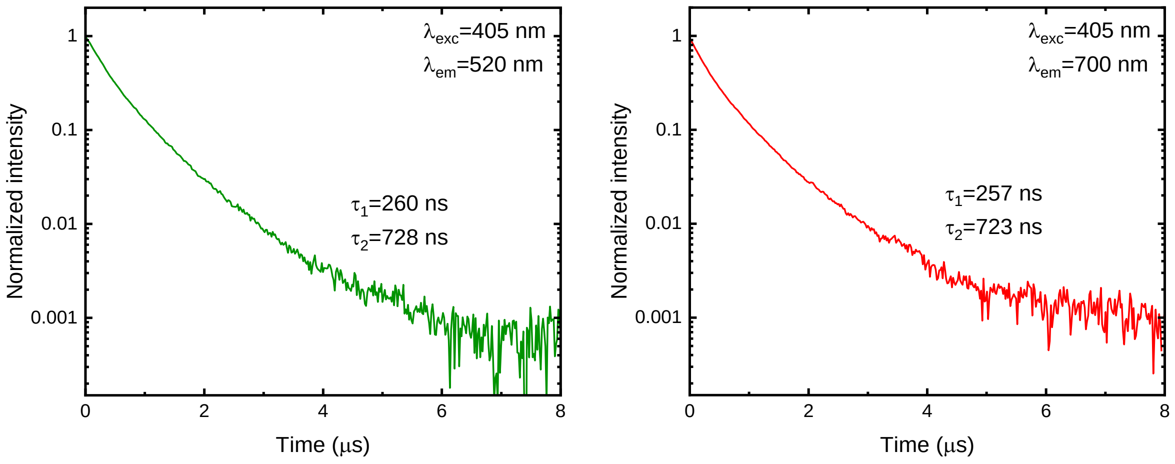

Figure S23. The luminescent decay profiles of $(Me_3Hy)[PbI_3]$ crystals measured at 80 K

Table S1. Crystal data and structure refinement for I and II phases of $(Me_3Hy)[PbI_3]$

	Phase II (230 K)	Phase I (375 K)
**Crystal data**		
Crystal system, space group	Hexagonal, $P6_3/m$	Hexagonal, $P6_3/mmc$
Temperature (K)	230(2)	375(2)
$a = b$, c (Å)	9.6202(3), 7.9298(2)	9.7735(5), 7.9594(3)
$\alpha = \beta$, γ (°)	90, 120	90, 120
V (Å^3)	635.57(4)	658.44(7)
Z / calculated density (g cm^{-3})	2 / 3.465	2 / 3.344
Absorption coefficient, μ (mm^{-1})	20.508	19.796
Index ranges	-11<=h<=12,<br>-12<=k<=11,<br>-9<=l<=9	-12<=h<=12,<br>-12<=k<=12,<br>-9<=l<=9
**Data collection**		
Theta range (°)	3.547 - 26.367	2.406° -26.301
No. of measured / independent reflections	4672 / 459	7706 / 283
R(int)	0.0275	0.0585
Completeness to theta = 25.242°	98.5 %	99.2 %
**Refinement**		
R [I > 2σ(I)], wR2 (I), S	0.0195, 0.0408, 1.129	0.0187, 0.0430, 1.147
Refinement method	Full-matrix least-squares on F^2	Full-matrix least-squares on F^2
Absorption correction	Gaussian	Gaussian
Data / restraints / parameters	459 / 6 / 32	283 / 9 / 26
Largest diff. peak and hole (e Å^{-3})	0.993, -0.727	0.384, -0.332
Extinction coefficient	0.0032(3)	0.0022(3)

For both phases M=663.03 g mol^{-1}; crystal size 0.139 x 0.112 x 0.083 mm.

Table S2. Geometric parameters (bond lengths (Å) and angles (°)) for phase I (HT) of (Me_3Hy)[PbI_3]

Pb1-I1#1	3.2361(6)	I1#2-Pb1-I1#5	93.850(12)
Pb1-I1#2	3.2361(6)	I1#3-Pb1-I1#5	86.150(12)
Pb1-I1#3	3.2361(6)	I1-Pb1-I1#5	86.150(12)
Pb1-I1	3.2361(6)	I1#4-Pb1-I1#5	180.0
Pb1-I1#4	3.2361(6)	Pb1-I1-Pb1#11	75.889(16)
Pb1-I1#5	3.2361(6)	N2#6-N1-N2#7	120.0
N1-N2#6	1.39(2)	N2#6-N1-N2	120.002(1)
N1-N2#7	1.39(2)	N2#7-N1-N2	120.002(1)
N1-N2	1.39(2)	N2#6-N1-C2#7	118.8(10)
N1-C2#8	1.47(2)	N2#7-N1-C2#7	16(6)
N1-C2#7	1.47(2)	N2-N1-C2#7	118.8(10)
N1-C2#9	1.47(2)	C2#8-N1-C2#7	122(2)
N1-C2#6	1.47(2)	N2#6-N1-C2#9	118.8(10)
N1-C2	1.47(2)	N2#7-N1-C2#9	16(6)
N1-C2#10	1.47(2)	N2-N1-C2#9	118.8(10)
N1-C1	1.50(3)	C2#8-N1-C2#9	113(5)
C2-H2A	0.9600	C2#7-N1-C2#9	32(10)
C2-H2B	0.9600	C2#8-N1-C2#6	32(10)
C2-H2C	0.9600	C2#7-N1-C2#6	113(5)
C1-H1A	0.9600	C2#9-N1-C2#6	122(2)
C1-H1B	0.9600	C2#8-N1-C2	122(2)
C1-H1C	0.9600	C2#7-N1-C2	113(5)
I1#1-Pb1-I1#2	86.151(12)	C2#9-N1-C2	122(2)
I1#1-Pb1-I1#3	93.849(12)	C2#6-N1-C2	113(5)
I1#2-Pb1-I1#3	180.00(2)	C2#8-N1-C2#10	113(5)
I1#1-Pb1-I1	180.0	C2#7-N1-C2#10	122(2)
I1#2-Pb1-I1	93.849(12)	C2#9-N1-C2#10	113(5)
I1#3-Pb1-I1	86.151(12)	C2#6-N1-C2#10	122(2)
I1#1-Pb1-I1#4	86.150(12)	C2-N1-C2#10	32(10)
I1#2-Pb1-I1#4	86.150(12)	N1-C2-H2A	109.5
I1#3-Pb1-I1#4	93.850(12)	N1-C2-H2B	109.5
I1-Pb1-I1#4	93.850(12)	H2A-C2-H2B	109.5
I1#1-Pb1-I1#5	93.850(12)	N1-C2-H2C	109.5
H2A-C2-H2C	109.5	H1B-C1-H1C	109.5
H2B-C2-H2C	109.5	N1-C1-H1A	109.5
N1-C1-H1A	109.5	N1-C1-H1B	109.5
N1-C1-H1B	109.5	H1A-C1-H1B	109.5
H1A-C1-H1B	109.5	N1-C1-H1C	109.5
N1-C1-H1C	109.5	H1A-C1-H1C	109.5
H1A-C1-H1C	109.5	H1B-C1-H1C	109.5

Symmetry transformations used to generate equivalent atoms:

#1 -x+2,-y,-z+1; #2 x-y,x-1,-z+1; #3 -x+y+2,-x+1,z; #4 y+1,-x+y+1,-z+1; #5 -y+1,x-y-1,z; #6 -x+y+1,-x+1,z; #7 -y+1,x-y,z; #8 -x+y+1,-x+1,-z+1/2; #9 -y+1,x-y,-z+1/2; #10 x,y,-z+1/2; #11 -x+2,-y,z-1/2

Table S3. Geometric parameters (bond lengths (Å) and angles (°)) for phase II (RT) of $(Me_3Hy)[PbI_3]$

Pb1-I1#1	3.2279(4)	I1-Pb1-I1#5	86.229(8)
Pb1-I1#2	3.2279(4)	I1#4-Pb1-I1#5	180.0
Pb1-I1#3	3.2280(4)	Pb1-I1-Pb1#8	75.780(11)
Pb1-I1	3.2280(4)	C1-N1-N2	54.9(9)
Pb1-I1#4	3.2280(4)	C1-N1-N2#6	104.0(16)
Pb1-I1#5	3.2280(4)	N2-N1-N2#6	120.002(1)
N1-C1	1.498(18)	C1-N1-N2#7	109.5(17)
N1-N2	1.56(7)	N2-N1-N2#7	120.000(19)
N1-N2#6	1.56(7)	N2#6-N1-N2#7	120.00(2)
N1-N2#7	1.56(7)	N2-N1-C2	20(5)
N1-C2	1.44(10)	N2#6-N1-C2	103(5)
C2-H2A	0.9700	N2#7-N1-C2	135(5)
C2-H2B	0.9700	N1-C2-H2A	109.5
C2-H2C	0.9700	N1-C2-H2B	109.5
N2-C1	1.41(4)	H2A-C2-H2B	109.5
N2-H4D	0.8572	N1-C2-H2C	109.5
N2-H4E	0.8867	H2A-C2-H2C	109.5
C1-H1A	0.9700	H2B-C2-H2C	109.5
C1-H1B	0.9700	C1-N2-N1	60(3)
C1-H1C	0.9700	N1-N2-H4D	112.3
I1#1-Pb1-I1#2	180.000(14)	N1-N2-H4E	94.3
I1#1-Pb1-I1#3	86.230(8)	H4D-N2-H4E	119.5
I1#2-Pb1-I1#3	93.770(8)	N2-C1-N1	65(3)
I1#1-Pb1-I1	93.771(8)	N2-C1-H1A	109.5
I1#2-Pb1-I1	86.229(8)	N1-C1-H1A	155.7
I1#3-Pb1-I1	180.0	N2-C1-H1B	109.5
I1#1-Pb1-I1#4	86.229(8)	N1-C1-H1B	55.7
I1#2-Pb1-I1#4	93.771(8)	H1A-C1-H1B	109.5
I1#3-Pb1-I1#4	86.229(8)	N2-C1-H1C	109.5
I1-Pb1-I1#4	93.771(8)	N1-C1-H1C	94.4
I1#1-Pb1-I1#5	93.771(8)	H1A-C1-H1C	109.5
I1#2-Pb1-I1#5	86.229(8)	H1B-C1-H1C	109.5
I1#3-Pb1-I1#5	93.771(8)		

Symmetry transformations used to generate equivalent atoms:

#1 x-y+1,x,-z+1; #2 -x+y+1,-x+2,z; #3 -x+2,-y+2,-z+1; #4 y,-x+y+1,-z+1; #5 -y+2,x-y+1,z; #6 -x+y+1,-x+1,z; #7 -y+1,x-y,z; #8 -x+2,-y+2,z-1/2

Table S4. Bond lengths (Å) of selected intermolecular contacts for phase II (RT) of $(Me_3Hy)[PbI_3]$

D - H···A	H···A (Å)	D···A [Å]	∠ D-H···A [°]
N(2) – H(4D) ···I(1) [-x+y,1-x,z]	3.260	3.494	98.63
C(1) – H(1A) ···I(1) [1-x,1-y,-1/2+z]	2.610	3.581	176.41
C(2) – H(2A) ···I(1)	3.190	3.808	122.95
N(2) – H(4D) ···I(1) [1-y,x-y,z]	3.210	3.883	137.19

Due to high symmetry, intermolecular interactions are multiplied by symmetry operations.

Table S5. Bond lengths (Å) of selected intermolecular contacts for phase I (HT) of (Me_3Hy)[PbI_3]

D - H···A	H···A (Å)	D···A [Å]	∠ D-H···A [°]
C(2) – H(2A) ···I(1) [x, x-y, 0.5-z]	2.947	3.809	150.15
C(2) – H(2B) ···I(1) [1-x+y, 1-x, z]	3.014	3.809	163.39

Due to high symmetry, intermolecular interactions are multiplied by symmetry operations.

Table S6. Crystal data obtained from PXRD experiment for phase III (LT) of $(Me_3Hy)[PbI_3]$

	**Phase III (190 K) PXRD**
Crystal system, space group	Orthorhombic, *Pbca*
Temperature (K)	190(2)
a , *b*, *c* (Å)	18.4083(3), 17.2246(4), 7.8215(2)
$\alpha = \beta$, γ (˚)	90, 90, 90
V (Å^3)	2480.02(5)
Z / calculated density (g cm^{-3})	8, 3.541
Absorption coefficient, µ (mm^{-1})	84.34
Theta range (°)	2 - 45
R_p, R_{wp}	0.0500, 0.0747

For phase III *M*=663.03 g mol^{-1}

Table S7. Geometric parameters (bond lengths [Å] and angles [°]) for phase III (LT) of (Me_3Hy)[PbI_3]

Pb1-I1	3.184	I2-Pb1-I3#1	87.59
Pb1-I2	3.229	I2-Pb1-I1#1	94.23
Pb1-I3	3.282	I2-Pb1-I2#1	88.50
Pb1-I2#1	3.225	I2-Pb1-I3#1	175.54
Pb1-I3#1	3.261	I3-Pb1-I1#1	88.21
Pb1-I1#1	3.287	I3-Pb1-I3#1	96.23
I1-Pb1-I2	86.28	I3-Pb1-I2#1	171.58
I1-Pb1-I3	89.62	I1#1-Pb1-I3#1	88.20
I1-Pb1-I1#1	177.75	I1#1-Pb1-I2#1	84.65
I1-Pb1-I3#1	91.44	I3#1-Pb1-I2#1	88.01
I1-Pb1-I2#1	97.56		

#1 x, 1.5-y, -0.5+z

Table S8. Bond lengths (Å) of selected intermolecular contacts for phase III (LT) of $(Me_3Hy)[PbI_3]$

D-H···A	H···A [Å]	D···A [Å]	∠ D-H···A [°]
C(3)-H(3B)···I(1) [x, 1.5-y, 0.5+z]	2.977	3.547	118.75
C(6)-H(6B)···I(1) [-0.5+x, 1.5-y, 1-z]	3.110	4.072	171.30
C(6)-H(6A)···I(1) [0.5-x, -0.5+y, 1+z]	3.038	3,835	140.45

Table S9. Assignment of IR and Raman bands observed for $(Me_3Hy)[PbI_3]$

$(Me_3Hy)[PbI_3]$ IR (cm^{-1})	Raman (cm^{-1})	Assignment
3311_{m}, 3247_{m}	3314_{vw}, 3251_{vw}	$\nu_{as}NH_2$
3169_{w}	3173_{vw}	$\nu_{s}NH_2$
3030_{w}, 3019_{w}	3032_{w}, 3020_{w}, 3013_{sh}	$\nu_{as}CH_3$
2946_{vw}, 2923_{vw}, 2852_{vw}, 2798_{vw}	2956_{w}, 2931_{w}, 2909_{vw}, 2880_{vw}, 2846_{vw}, 2800_{vw}	$\nu_{s}CH_3$
1600_{m}	1600_{vw}	δNH_2
1468_{vs}, 1454_{m},	1470_{vw}, 1454_{sh}	$\delta_{as}CH_3$
1439_{m}, 1432_{sh}, 1405_{w},	1436_{w}, 1407_{vw}	$\delta_{s}CH_3$
1374_{vw}	1375_{vw}	ωNH_2
1273_{w}	1276_{vw}, 1251_{vw}	τNH_2
1251_{vw}, 1203_{vw}, 1178_{vw}, 1135_{w}, 1039_{m}	1206_{vw}, 1136_{vw}, 1106_{vw}, 1041_{vw}	ρCH_3
942_{m}	945_{w}	νCN
889_{m}	891_{vw}	ρNH_2
750_{w}, 737_{w}	753_{w},739_{w}	νNN
475_{w}, 457_{vw}	476_{vw}, 459_{vw}	δCNC
373_{w}	375_{vw}	τCH_3
291_{w}, 216_{w}	291_{vw}	τCH_3
105_{s}, 80_{s}	101_{vs}, 93_{sh}	νPbI
	60_{m}	$\delta PbI+L(PbI_6)$

Key: ν, stretching (s, symmetric; as, antisymmetric); δ, bending (s, symmetric; as, antisymmetric); ρ, rocking; ω, wagging; τ, twisting; vs, very strong; s, strong; m, medium; w, weak; vw, very weak

Table S10. Raman pressure intercepts (ω_0) and coefficients (α) for three phases of $(Me_3Hy)[PbI_3]$ together with proposed assignment

Mode no	AP phase		HPI phase		HPII phase		Assignment
	ω_0 (cm^{-1})	α=dω/dP ($cm^{-1}GPa^{-1}$)	ω_0 (cm^{-1})	α=dω/dP ($cm^{-1}GPa^{-1}$)	ω_0 (cm^{-1})	α=dω/dP ($cm^{-1}GPa^{-1}$)	
1			132.6	8.61			νPbI
2			111.1	8.49			νPbI
3	101.4	5.52	99.5	2.08	96.2	2.49	νPbI
4			89.7	2.02	93.8	1.22	νPbI
5			74.8	1.98			δPbI+L(PbI_6)
6			72.6	-1.38			δPbI+L(PbI_6)
7	66.0	0.88	60.9	2.75	61.1	0.37	δPbI+L(PbI_6)
8			44.3	2.83			δPbI+L(PbI_6)

Key: ν, stretching; δ, bending; L, libration
